# Mathematical Philology: Entropy Information in Refining Classical Texts' Reconstruction, and Early Philologists' Anticipation of Information Theory

**DOI:** 10.1371/journal.pone.0008661

**Published:** 2010-01-13

**Authors:** John L. Cisne, Robert M. Ziomkowski, Steven J. Schwager

**Affiliations:** 1 Department of Earth and Atmospheric Sciences, Cornell University, Ithaca, New York, United States of America; 2 Department of Modern Languages and Literatures, Ithaca College, Ithaca, New York, United States of America; 3 Department of Biological Statistics and Computational Biology, Cornell University, Ithaca, New York, United States of America; University of East Piedmont, Italy

## Abstract

Philologists reconstructing ancient texts from variously miscopied manuscripts anticipated information theorists by centuries in conceptualizing information in terms of probability. An example is the editorial principle *difficilior lectio potior* (DLP): in choosing between otherwise acceptable alternative wordings in different manuscripts, “the more difficult reading [is] preferable.” As philologists at least as early as Erasmus observed (and as information theory's version of the second law of thermodynamics would predict), scribal errors tend to replace less frequent and hence entropically more information-rich wordings with more frequent ones. Without measurements, it has been unclear how effectively DLP has been used in the reconstruction of texts, and how effectively it could be used. We analyze a case history of acknowledged editorial excellence that mimics an experiment: the reconstruction of Lucretius's *De Rerum Natura*, beginning with Lachmann's landmark 1850 edition based on the two oldest manuscripts then known. Treating words as characters in a code, and taking the occurrence frequencies of words from a current, more broadly based edition, we calculate the difference in entropy information between Lachmann's 756 pairs of grammatically acceptable alternatives. His choices average 0.26±0.20 bits higher in entropy information (95% confidence interval, *P* = 0.005), as against the single bit that determines the outcome of a coin toss, and the average 2.16±0.10 bits (95%) of (predominantly meaningless) entropy information if the rarer word had always been chosen. As a channel width, 0.26±0.20 bits/word corresponds to a 0.790.79^+0.09^_−0.15_ likelihood of the rarer word being the one accepted in the reference edition, which is consistent with the observed 547/756 = 0.72±0.03 (95%). Statistically informed application of DLP can recover substantial amounts of semantically meaningful entropy information from noise; hence the extension *copiosior informatione lectio potior*, “the reading richer in information [is] preferable.” New applications of information theory promise continued refinement in the reconstruction of culturally fundamental texts.

## Introduction

How accurately have culturally fundamental texts been transmitted to the present by way of variously miscopied manuscripts? If the accuracy can be measured, can it be improved, and if so, how? Philology traditionally has been concerned almost entirely with information of the semantic kind, that is, with meaning. Here we are concerned instead with what has been called entropy information, information entropy, and Shannon entropy (and sometimes negentropy in recognition that a higher information content corresponds to a higher degree of disorder). In the first study of its kind, we measure the accuracy of transmission in bits/word of meaningful entropy information. The case in point is one of acknowledged editorial excellence and cultural importance: the reconstruction of Lucretius's *De Rerum Natura*, beginning with Lachmann's 1850 edition [1], a defining example of modern textual criticism [2]–[4].

### 1. Anticipation of Information Science by Early Philologists

#### 1.1. Information, randomness, and probability

Information theory originated in twentieth-century telecommunications engineering, as is well known [5]–[8], but it has a long and apparently unappreciated prehistory in philology. Theorizing about how best to recover accurate messages from noisy signals goes back many centuries to scholars who endeavored to reconstruct ancient texts from variously miscopied manuscripts. Systematically organized, institutionally sponsored comparison of manuscripts expressly for this purpose dates back at least to the founding of the Library at Alexandria (∼300 BCE), if not to far older Mesopotamian clay-tablet libraries [9].

Beginning with notions developed independently by Wiener, Shannon established that information is a probabilistic phenomenon closely akin to entropy; that information entropy tends to be lost as noise during transmission in a manner analogous to the increase in physical entropy according to the second law of thermodynamics; and that the losses are recoverable from noise, sometimes completely, from redundancies in the information received [7], [10], [11]. Scholars centuries before had intuited enough about information as orderedness to develop a remarkably similar probabilistic approach to recovering original text from corrupt copies. Medieval scribes recognized that copying error has a random or chaotic element, and even invented a counterpart to Maxwell's Demon as its source: Tutivillus, whom they adopted as their patron demon [12]. (The demon is commonly known as Titivillus as well as Tutivillus — names which, appropriately enough, must be misspellings of one another. “Tutivillus” is used here because it is preferred in the definitive work [12].)

#### 1.2. The difficilior lectio potior principle (DLP)

In associating information with probability, philologists at least as early as Erasmus (?1466–1536) [3], and perhaps even as early as Probus (first century CE) [4], recognized that when scribes mistakenly substitute one wording for another, they tend to simplify, to replace less common forms with more common ones (the utrum in alterum abiturum erat principle) [3]. From this follows the editorial principle difficilior lectio potior (DLP): all else being equal, “the more difficult reading [is] preferable” [3], [13], [14] or “the less probable reading [is] preferable” [15]. The same basic idea is known in New Testament philology as the proclivi scriptioni praestat ardua principle (“The difficult is to be preferred to the easy reading”) [16].

As a statistical generalization, DLP is well grounded. Consider an author's original manuscript (autograph copy) of a text containing N = *n*(1)+*n*(2)+…+*n*(*k*)+…+*n*(*L*) words belonging to *L* lemmata. Let us consider first the ideal case of an indefinitely long message (that is, N → ∞) in which each lemma *k* occurs with probability *p*(*k*). Treating each lemma as a character in a code, the information content per character of the message will be
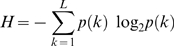
(1)


As Shannon showed [5], [6], random replacement of a word of lemma *i* with a word of lemma *j* tends to reduce the information content per character *H* unless the occurrences of lemmata *i* and *j* are statistically independent of one another. Let *H*(*x*) and *H*(*y*) be respectively the information entropies of the original text (message *x*) and the copied text (message *y*), let *p*(*i,j*) be the probability of the event that lemma *i* in the original has been replaced by lemma *j* in the copy, and let *H*(*x,y*) be the entropy of the joint occurrence of *x* and *y*:
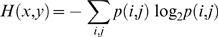
(2)


It can be shown that the total amount of information in the two manuscripts collectively, *H*(*x,y*), is no more than the sum of the information in the two manuscripts individually, *H*(*x*)+*H*(*y*): *H*(*x,y*)≤*H*(*x*)+*H*(*y*) (5, 6). Information will be lost unless the occurrences of all lemmata *i* and *j* are statistically independent, that is, *p*(*i,j*) = *p*(*i*) *p*(*j*), which implies that the occurrences of *i* and *j* are uncorrelated. This generalization about information entropy corresponds to the second law of thermodynamics. In statistical mechanics, the condition *H*(*x,y*) = *H*(*x*)+*H*(*y*) corresponds to a reversible process and conservation of entropy, whereas *H*(*x,y*)<*H*(*x*)+*H*(*y*) corresponds to an irreversible process and increase in entropy.

Because correlation in the co-occurrence of words and symbols is a characteristic of human language, copying error will tend to result in information loss [5], [6], [7], [10], [11]. Correlation can take many forms. Redundancy, one form, is discussed in section 2.2 below. Particularly strong correlation is to be expected in cases to which DLP applies, because the condition that the alternative words be more or less equally acceptable will drastically limit possible co-occurrences.

Thus Tutivillus, like Maxwell's Demon, is a sorting demon with respect to entropy, but unlike its counterpart, has a dual nature as a randomizing demon with respect to semantic information. Whereas Maxwell's Demon decreases physical entropy by intelligently sorting gas molecules by energy level (which requires information about their energy levels), Tutivillus decreases information entropy by playing perversely on words' correlated co-occurrence.

Let us turn now to finite messages because it is to these that DLP applies. Consider a message so long that the relative abundance *n*(*k*)/N of each lemma *k* approximates its probability of occurrence, *p*(*k*) (implying *n*(*k*)≫1, since 1/N is likely an inaccurate approximation). It is found from equation (1) that the frequency-weighted geometric mean 〈*p*〉 of the probabilities *p*(*k*) directly reflects the information entropy *H* of the message [5], [6]: 〈*p*〉≈2*^–H^*. This applies to the weighted geometric mean word frequency 〈*n*〉 = 〈*p*〉 N as well: 〈*n*〉≈N · 2*^−H^*. If words in an original message (*x*) are substituted one-for-one with words in the copy (message *y*), so that N remains constant, the weighted mean frequency in the copy, 〈*n* (*y*)〉, is related as follows to the corresponding frequency in the original, 〈*n* (*x*)〉, by the difference Δ*H* = *H*(*y*) – *H*(*x*) in the information content per word:
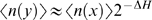
(3)


If, as expected, Δ*H*<0 (presuming that variation in abundances *n*(*k*) in the original message leaves information to be lost), the *utrum in alterum abiturum erat* principle follows as a consequence: 〈*n* (*y*)〉>〈*n* (*x*)〉, which is to say that, when mistakes occur, less common words tend to be replaced by more common ones [3]. From this follows the *difficilior lectio potior* principle (DLP) that, to recover the information lost, an editor does well to choose the less common of two equally acceptable alternative words as more likely the author's original.

Thus there is no question that DLP *ought* to work. The question is *how well* it works.

#### 1.3. Historical note on entropy awareness and C.P. Snow's “Two Cultures”

**Figure 1:**
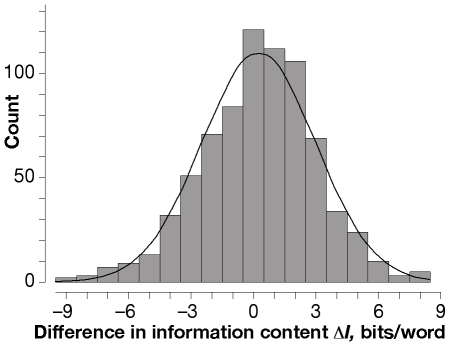
The difference Δ*I* in entropy information between 756 pairs of otherwise acceptable alternative words in the two manuscripts on which Lachmann based his reconstruction of Lucretius's *De Rerum Natura* (*On the Nature of Things*, ∼60 BCE) [1], and a Gaussian curve fitted to the data. The mean value 〈Δ*I*〉 = +0.257±0.196 bits/word (95% confidence interval; *P*–value = 0.005, one-sided) corresponds to a 0.79^+0.09^_−0.15_ likelihood of the rarer word being the better choice, showing the value of the *difficilior lectio potior* principle (DLP) that “the less probable reading is preferable” in choosing between otherwise acceptable alternatives in reconstructing a text from variously miscopied manuscripts. The Renaissance and earlier philologists who framed DLP evidently had a prescient understanding of information as a probabilistic phenomenon.

C.P. Snow made awareness of the second law of thermodynamics his litmus test for dividing academics into his famous Two Cultures, humanistic and scientific [17]. Centuries before probability theory, philologists — quintessential humanists — had an intuitive understanding of the second law as it applies to information, as we document further below. Had Karl Friedrich Gauss not been turned from an intended career in philology by his discovery of the geometrical constructability of the regular 17-gon and related implications for number theory [18], the results of Snow's litmus test might not have been so sharp. As Figure 1 shows, Gauss could even have discovered the Gaussian distribution in philological rather than astronomical data.

### 2. The Process of Reconstructing a Text

#### 2.1. “Lachmann's Method”

Ever since Erasmus, if not before, the favored approach to reconstructing a text has been first to reconstruct the “family tree” (stemma) of manuscripts based on the occurrence of major “mutations” (characteristic errors) [4]. Current methods have grown up around the one established by Karl Lachmann (1793–1851), the founder of modern textual reconstruction (textual criticism) [14]. “Lachmann's method,” as the general approach has come to be known, is essentially the cladistic method developed independently a century later by taxonomists for attempting to establish the relative recency of common descent among organisms [19]–[22].

The steps in preparing a new edition are: identifying and studying comparatively the surviving manuscripts of the text (exemplars); identifying the characteristic errors that appear to distinguish the major branches of the stemma; reconstructing the stemma in detail by seeking the tree that accounts most parsimoniously for the occurrence of characteristic errors in terms of the relative recency of common descent among exemplars; selecting for further analysis only those readings evidently closest to the author's original, and eliminating from further consideration those variants that contain no additional information; collating the selected manuscripts word by word; and finally, choosing among the alternative wordings in the effort to reconstruct the closest possible approximation to the original text, footnoting the rejected alternatives in the new edition's *apparatus criticus* [3], [14]. DLP figures in the final step when alternatives are more or less equally acceptable.

In its strictest form, Lachmann's method assumes that the manuscript tradition of a text, like a population of asexual organisms, originates with a single copy; that all branchings are dichotomous; and that characteristic errors steadily accumulate in each lineage, without “cross-fertilization” between branches [13]. Notice again the awareness that disorder tends to increase with repeated copying, eating away at the original information content little by little. Later schools of textual criticism relax and modify these assumptions, and introduce more of their own [4], [14].

#### 2.2. Decisions between single words

Many types of scribal error have been catalogued at the levels of pen stroke, character, word, and line, among others [3], [13], [14]. Here we limit ourselves to errors involving single words, for it is to these that DLP should apply least equivocally. This restriction minimizes subjective judgments about one-to-one correspondences between words in phrases of differing length, and also circumvents instances in which DLP can conflict with a related principle of textual criticism, *brevior lectio potior* (“the shorter reading [is] preferable”) [4].

Limiting ourselves to two manuscripts with a common ancestor (archetype), let us suppose as before that wherever an error has occurred, a word of lemma *j* has been substituted in one manuscript for a word of the original lemma *i* in the other. But can it be assumed realistically that the original lemma *i* persists in one manuscript? The tacit assumption is that errors are infrequent enough that the probability of two occurring at the same point in the text will be negligible, given the total number of removes between the two manuscripts and their common ancestor. For instance, in the ∼50,000-word text of Lucretius, we find 2,095 variants denoting errors of one sort or another in two manuscripts that, as Lachmann and others have conjectured, are each separated at two or three removes from their most recent common ancestor. At least for ideologically neutral texts that remained in demand throughout the Middle Ages, surviving parchment manuscripts are unlikely to be separated at very many more removes, because a substantial fraction (on the order of 10% in some instances) can survive in some form [23], [24], contrary to anecdotally based notions that only an indeterminately very much smaller fraction remains [25]–[27].

Let us suppose further that copying mistakes in a manuscript are statistically independent events. The tacit assumption is that errors are rare and hence sufficiently separated to be practically independent in terms of the logical, grammatical, and poetic connections of words. With Lachmann's two manuscripts of Lucretius, the ∼2,100 variants in ∼50,000 words of text correspond to a net accumulation of about one error every four lines in Lachmann's edition in the course of about five removes, or of roughly one error every 20 lines by each successive scribe. The separation of any one scribe's errors in this instance seems large enough to justify the assumption that most were more or less independent of one another.

Finally, let us suppose that an editor applying DLP chooses the author's original word of lemma *i* with probability *p*, and the incorrect word of lemma *j* with probability 1 – *p*. Under these conditions, the editor's choice amounts to a Bernoulli trial with probability *p* of “success” and probability 1 – *p* of “failure.” But how can it be assumed that *p* is constant among all words when any given *k*th lemma in a manuscript will be unique, and hence should have its own characteristic probability *p_k_* of being correctly copied? Assuming that *p* is constant among lemmata amounts to assuming that the *p_k_*s approach a common value *p* as an average, for which justifications can be found in instances like this one [28]. That is, given a large number of choices among a large number of lemmata, the law of averages will apply, and, for practical purposes, all choices could just as well have been governed by a constant probability *p*.

Under these conditions, the editor's probability *p* of choosing correctly relates directly to the amount of pertinent information entropy 0≤*h*≤1 in bits/choice *unavailable* to guide editorial decisions, and equation (1) takes the form:
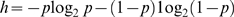
(4)


As equation (4) shows, a single bit of information entropy suffices to predict correctly the outcome of a Bernoulli trial (*h* = 0 bit, *p* = 1 or, for a contrarian choice, *p* = 0). The amount of *non-redundant* information entropy per choice, the channel width *c*, corresponds to the amount that reaches the editor [6], [7]:
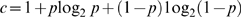
(5)


Redundancy is possible, which corresponds to the situation *c*>1 bit/word, which ensures *p* = 1. In this case, DLP would be literally too good to be true: word frequency alone would suffice for a correct choice, *independent of context and semantic content.*

#### 2.3. Evaluating a reconstructed text

What evidence is there that earlier philologists ever paid anything more than lip service to DLP, and that they indeed understood enough about information in the sense of entropy to recapture measurable amounts of it? Given a suitable text against which to judge the correctness of choices between alternative words, DLP becomes a testable hypothesis. The ideal standard of comparison is the archetype of the manuscripts being used to reconstruct the text. A problem is immediately apparent: an ideal test would be possible only in the seldom if ever realized case in which the archetype has been unequivocally identified subsequent to the reconstruction of its text; for if the archetype were already known, what incentive would there be to reconstruct it? Thus for testing DLP, we must be content with evaluating an earlier, more narrowly based edition against later, more broadly based editions. Ideally, all the editions would be statistically independent of one another, but this is exceedingly unlikely.

We need to test statistically whether the probability *p* in equations (4) and (5) is greater than 0.5, the probability of correctly calling a toss of a fair coin. We can do this by testing whether two estimated values of *p* are significantly greater than 0.5: the first is the estimate *P_1_* found numerically from an estimate of *c* in equation (5) as the average amount of information gained or lost in some large number of decisions; the second is *P_2_*, the fraction of decisions that are correct. If both tests support the alternative hypothesis *p*>0.5, there is reason to conclude that DLP is valid.

But why be concerned with information at all if DLP maintains simply that an editor will more often be correct in choosing the less common of equally acceptable alternative words? As will be explained, it is quite possible for an editor to choose correctly by selecting the less common word more often than not, thereby satisfying DLP (*P_2_*>0.5), and yet lose much more information than would be lost in making decisions by coin toss (c≤0.5 bits/word because, in sum, incorrect choices lost more information than correct choices gained), implying *P_1_*<0.5 and thus contradicting DLP.

Let us turn now to the case of an archetype whose text contains N = *n*(1)+*n*(2)+…+*n*(*k*)+…+*n*(*L*) words belonging to *L* lemmata. Treating each lemma as a character in a code, as before, the information content *I* (*x*) of the archetype's text (message *x*) is
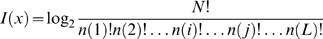
(6)


The expression on the right is the logarithm of the multinomial probability of the particular set of numbers *n*(*k*) occurring by chance. *H*(*x*) in equation (1) is the limit as N → ∞ of the average *I*(*x*)/N as found by applying Stirling's approximation to the factorials in equation (6). The probabilities *p*(*k*) in equation (1) correspond to the relative abundances *n*(*k*)/N. If equation (1) were used as an approximation in place of the exact equation (6), the probabilities *p*(*k*) would have to be estimated separately from some sample of the language. Equation (6) avoids this difficulty. At the same time, it more accurately assesses the substantial information content of rare words, which is important because in general most occur quite infrequently. For instance, in Lucretius's *De Rerum Natura*, ∼4,500 lemmata are represented in the ∼50,000-word text, and of these, ∼1,600 occur only once.

Suppose now that a copyist has mistakenly replaced an original word of lemma *i* with an otherwise equally acceptable word of lemma *j* at some point in the text. All else remaining the same, the information content *I* (*y*) of the corrupt copy (message *y*) will be
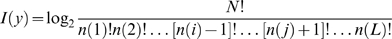
(7)and the apparent change in information content Δ*I* = *I*(*y*) – *I*(*x*) will be
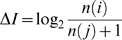
(8)


Questions about expression (8) in relation to continuous as opposed to discrete information are taken up in section 2.4 below.

The average of Δ*I*-values throughout the text, 〈Δ*I*〉, corresponds to *c* in equation (5). Notice that *n*(*i*)≥1 because, by hypothesis, the original lemma *i* is one of the possibilities. Notice also that Δ*I* can be positive, negative, or zero. A copying mistake may lose semantic information, but it can either increase or decrease the amount of entropic information.

Whenever a copying error is made, an amount of information |Δ*I*| given by equation (8) is cast in doubt. Reconstruction of a text can be viewed as a process of recovering as much of this information is possible. Wherever the editor endeavors to correct a mistake, choosing the correct lemma *i* will add the amount of information –Δ*I* from equation (8), and choosing the incorrect lemma *j* will add the amount +Δ*I*. If the editor always chooses the less frequent word, a non-negative amount of information |Δ*I*| will be added each time.

The firmest prediction for testing DLP comes from the second law as it applies to information: if the editor has successfully taken advantage of entropy information, then the average Δ*I*-value for a large number of binary decisions should be distinctly greater than zero, that is, 〈Δ*I*〉>0 bits/word. How much greater than zero will depend on many factors, such as the language itself, the author's vocabulary, each scribe's attention span, the editor's competence, and the psychologies of all involved. In itself, 〈Δ*I*〉 significantly greater than 0 bits/word constitutes *prima facie* evidence that DLP applies to the reconstructed text, because 〈Δ*I*〉>0 bits/word implies by way of equation (5) that the editor has a distinctly higher likelihood *p* of choosing correctly by choosing the less common word than by flipping a coin (that is, *p*>0.5). On the other hand, DLP would not apply if 〈Δ*I*〉≤0 bits/word; words' frequencies of occurrence *n*(*k*) then could be said to have provided, if anything, entropy *disinformation*.

There is no doubt that editorial decisions are based primarily on semantic information. Hence there is reason to believe that entropic information ordinarily contributes less than half of the single bit needed to decide a binary choice, especially since DLP comes into play only when there is enough non-entropic information to establish that both alternatives are acceptable, and more or less equally so. Thus we have a second expectation: that 〈Δ*I*〉 is probably less than 0.5 bits/word. A 〈Δ*I*〉-value even approaching 1 bit/word would appear practically impossible, like the case of *c*>1 bit/word in equation (5), as it would imply that the correct word generally could be chosen on the basis of frequency alone.

All that can be estimated from 〈Δ*I*〉 alone is the maximum amount of entropy information that *could* have contributed to the single bit needed for a successful decision. The problem in establishing how much the entropy information actually *did* contribute to the editor's decision is the inherent redundancy of language itself, typically ∼50–75% in modern printed English [10]. The question is whether the editor tended to dismiss actually meaningful entropic information as redundant.

Evidence comes by way of equation (8). If 〈Δ*I*〉≥0, the (non-redundant) entropy information corresponds to a channel width 0≤*C_1_*≤1 analogous to channel width *c* in equation (5); if 〈Δ*I*〉<0, there is no corresponding channel width *C_1_*. If 〈Δ*I*〉≥0 bits/word, the probability *P_1_* corresponding to *p* in (8) can be found numerically from *C_1_*; if 〈Δ*I*〉<0, there is no corresponding probability *P_1_*. Now *p* can also be estimated as the fraction of editorial choices *P_2_* that agree with the archetype or its stand-in. Notice that *P_2_* depends only on the total number of the editor's successful choices, whereas *P_1_* depends primarily on the distribution of the frequency of occurrence of words as reflected in the distribution of Δ*I*-values (Figure 1). Though not independent of one another, *P_1_* and *P_2_* could differ substantially. If *P_1_*≈*P_2_* within the range of uncertainty, evidence then supports the conclusion that the editor has indeed taken entropic information into account.

To sum up, 0<〈Δ*I*〉<1 bit/word supports the conclusion that entropic information contributed to the editor's decisions, and hence that DLP applies to the edition. If *P_1_*≈*P_2_*, the conclusion is reinforced, as it is if 〈Δ*I*〉<0.5 bits/word. If the conclusion holds, then the prediction from the second law is confirmed, and DLP follows as a consequence. Though DLP concerns the frequency of alternative words *relative to the total number*, the real test of DLP is the frequency of alternative words *relative to one another*, which is the quantity that determines the difference in entropic information, as equation (8) shows.

#### 2.4. Discussion

Would the corresponding expression derived from equation (1), Δ*I* = log_2_ [*p*(*i*)/*p*(*j*)]≈log_2_ [*n*(*i*)/*n*(*j*)], be preferable to equation (8): Δ*I* = log_2_ {*n*(*i*)/[*n*(*j*)+1]}? This cannot be the case: in 95 out of 756 choices between acceptable alternative words in reconstructing Lucretius's *De Rerum Natura*, *n*(*j*) = 0, giving a meaningless Δ*I* → log_2_ [*n*(*i*)/0] each time.

How could a text approach the theoretical minimum-information condition *I* = 0 bits in which all words belong to a single lemma, when equation (8) allows the introduction of previously unrepresented lemmata, that is, ones with *n*(*j*) = 0? A text may gain or lose lemmata through repeated miscopying, but as equation (3) shows, the overall trend will be toward replacement of less common lemmata by more common ones, with the eventual loss of lemmata from the text. Is this a realistic possibility to consider in a manuscript only one or a few removes from its archetype? Loss during copying should be common because most lemmata occur quite infrequently. With Lucretius's *De Rerum Natura*, for instance, ∼1,600 out of ∼4,500 lemmata in the archetype of manuscripts *O* and *Q* apparently occurred only once (*n*(*i*) = 1) and hence would have been on the verge of extinction at the very first copying.

## Results

### 1. Lachmann and Lucretius

We analyze Lachmann's 1850 reconstruction of Lucretius's *On the Nature of Things* (*De Rerum Natura*, ∼60 BCE) [1], which he based on two ninth-century manuscripts, known as *Oblongus* (*O*) and *Quadratus* (*Q*) (the two oldest then known), plus a fifteenth-century manuscript (*L*) that he took to have descended independently from a common ancestor, even though all its scribal variants seem to be found in either *O* or *Q*. It is now generally accepted that *L* and all other fifteenth-century Italian manuscripts are descended from *O*, so that, for practical purposes, Lachmann based his edition on *O* and *Q* alone [2], [29]–[31]. It is also generally accepted on the basis of paleographic and codicological evidence that *O* and *Q* are both descended at one or two removes from a lost ancestor known as ω^II^, and that ω^II^ in turn is twice removed from a lost fourth- or fifth-century ancestor known as Ω [2], [30].

### 2. Differences in Information Entropy between Manuscripts

We evaluate Lachmann's reconstruction using the later and much more broadly based reconstruction by Ernout [32] as a stand-in for the archetype, and using Govaerts's [33] tabulation of word frequencies in Ernout's edition. Govaerts's data are of a type seldom collected, and are the only such data available on Lucretius. The fifth edition of Martin [34] is used for comparison. Like Ernout's edition, Martin's has long been one of the standards.

Ernout's text contains N = 49,658 words belonging to *L* = 4,492 lemmata [33], and is found from equation (6) to have an entropy information content of *I*≈474 ·10^3^ bits (∼58KB). The entropy information per word, *H* = *I*/N≈9.54 bits/word, is comparable to the 9–12 bits/word in present-day written English when calculated in the same manner [10], [11].

We count 2,095 instances in which Lachmann's *apparatus criticus* [1] lists one or more words as alternatives for one or more others (see Table S1). Some of the discrepancies are easily correctable errors; for instance, ones of spelling, syntax, or repetition. Some involve whole phrases. Some may be due to different editors' alternative readings of the same letters in the same manuscript. Here we analyze only those instances to which DLP should apply unequivocally: 756 cases involving single, correctly spelled words that are easily seen to correspond one-to-one between *O* and *Q*, and that both Ernout and Martin accept as the correctly read alternatives (out of 830 on which only Lachmann and Ernout agree).

We calculate the entropy difference Δ*I* between Lachmann's two alternative words as the difference resulting from the substitution of each one into Ernout's text according to equation (8). For instance, in Book III, line 1038, the alternatives are *potitus* (“acquired,” *n* = 6) and *potius* (“better” or “preferable,” *n* = 23) for an absolute difference in entropy information of | Δ*I*| = |log_2_ [6/(23+1)]| = 2.0 bits to be gained or lost. We take Ernout's text as establishing the correct alternative, as if it were the text of the common ancestor ω^ II^. In this instance, Lachmann chose *potitus*, as did Ernout and Martin, thereby recovering Δ*I* = +2.0 bits that otherwise would have been lost to noise. Notice that of the 2.0 bits, 1.0 bit is redundant, which would imply any editor should have more than enough entropy information to choose correctly between semantically equivalent alternatives. Consistent with this, all three editors made the same choice.

The distribution of Δ*I*-values for all 756 instances is nearly Gaussian (Figure 1). The mean difference in entropy information is 〈Δ*I*〉 = +0.257±0.196 bits/word (95% confidence interval; the observed significance level or *P*–value = 0.005, one-tailed because the second law gives reason to believe the population mean of Δ*I* is positive, so the alternative hypothesis is that this population mean is greater than zero). This is contrasted with the 1 bit/word needed to determine the outcome of a Bernoulli trial, and the average 2.161±0.095 bits/word (95% confidence interval) of predominantly meaningless information that would have been added if the editor had chosen the rarer word in all 756 cases.

Similar results were obtained with the 830 instances in which Lachmann and Ernout, but not necessarily Martin, agree on the alternative lemmata: 〈Δ*I*〉 = +0.292±0.187 bits/word (95% confidence interval; *P*–value = 0.001, one-tailed), and with subsamples in which the rarest lemmata were eliminated (a notable point because some of these are best known from Lucretius's poem).

As a channel width, 0.26±0.20 bits/word (in significant figures) corresponds by way of equation (5) to a *P_1_* = 0.79^+0.09^_−0.15_ likelihood of the rarer word being correct, in agreement with the *P_2_* = 547/756 = 0.72±0.03 (95% confidence interval) fraction of Lachmann's choices taken to be correct by Ernout. Similar results were obtained uniformly with additional data sets, beginning with the set of all 830 cases in which Lachmann and Ernout but not necessarily Martin agree on the alternative reading, and including various subsets of those 830 cases.

The implication from 0<〈Δ*I*〉<1 bit/word, reinforced by *P_1_*≈*P_2_*, is that Lachmann recovered a substantial and realistic amount of semantically meaningful entropic information, and hence that DLP applies to his reconstruction. Lachmann evidently found it possible to increase the odds of choosing correctly between more or less equally acceptable alternatives from 0.5 for a fair coin toss to about 0.7–0.8 (0.79^+0.09^_−0.15_, 0.72±0.03), on average.

## Discussion

Our results suggest that the *difficilior lectio potior* principle (DLP) can indeed be useful as an editorial rule of thumb. This is consistent with the notion that the early philologists who framed DLP had prescient understanding of information as a probabilistic phenomenon.

The results also suggest an extension of DLP as a quantitatively testable hypothesis: *copiosior informatione lectio potior*, “the reading richer in [entropic] information [is] preferable.” Conclusively testing this hypothesis will require analysis of the manuscript traditions of many more texts.

The results call attention to the mathematical nature of philology, and to its connections with information science. They suggest that applications of information theory, particularly statistical aspects developed to high levels of sophistication in cryptography [35], could prove valuable in continuing to refine the reconstruction of culturally fundamental texts.

## Materials and Methods

### 1. Data

Table S1 gives the data used in this study.

### 2. Issues of Latinity

In the attempt to estimate each word's entropy information as objectively and unambiguously as possible, we treat grammatically justifiable words without regard to inflection, context, and semantic content (meaning); and we calculate entropy information by treating each word's lemma as if it were a symbol. If inflection or association in context were taken into account, it often would be impossible to classify an individual Latin word uniquely as belonging to one and only one symbol, and thus impossible to associate that word uniquely with a definite amount of information. For instance, the noun *feminae* could be genitive or dative singular, or nominative plural, the correct choice depending on the reader's interpretation of the word's sometimes ambiguous relationship to others in the sentence.

Taking all of the inflections of a word like *femina* as representing a single symbol avoids many ambiguities, but at certain costs. One of these is losing whatever information is contained in any one word's contextual association with others in a sentence. Another is losing whatever information is contained in the distinction between lemmata of the same spelling. The word *cum*, for instance, can be read as either a conjunction or a preposition, the choice again depending on the reader's assessment of the context. Where a word such as *cum* could represent more than one part of speech (that is, more than one lemma), we count it as belonging to all possible lemmata and reckon its frequency of occurrence accordingly.

Although the *Oxford Latin Dictionary* [36] is perhaps more widely known, we chose Lewis and Short's *A Latin Dictionary* [37] as our standard of reference because it is favored by the Pope's principal Latinist, Reginald Foster [38]. Also, we accept as correct well-known medievalisms, such as *que* (not the enclitic *-que*) for *quae*, that occur in Lachmann's *apparatus criticus* [1].

## Supporting Information

Table S1 The 2,095 textual variants we note in the apparatus criticus for Karl Lachmann's 1850 edition of Lucretius's De Rerum Natura [1].
(0.17 MB PDF)
