# Supplementary material for: Mathematical Philology: Entropy Information in Refining Classical Texts' Reconstruction, and Early Philologists' Anticipation of Information Theory
Source: PLoS One. 2010 Jan 13;5(1):e8661. doi: 10.1371/journal.pone.0008661 (PMC2800184; doi:10.1371/journal.pone.0008661)
Supplement: Table S1 — The 2,095 textual variants we note in the apparatus criticus for Karl Lachmann's 1850 edition of Lucretius's De Rerum Natura [1]. (0.17 MB PDF) [file pone.0008661.s001.pdf]

Book	line	Lachmann word frequency	Alternate word frequency	Ernout word frequency	Lachmann word	Alternate word	Ernout word	Remarks
1	16	.	1	15	pergis	tergis	pergis	ok
1	27	.	.	.	ornatum (3)	oralatum		sp
1	32	.	.	.	moenera	monera		sp
1	33	.	0	5	regit [, in] (5)	regium [in] (0)		NB
1	35	.	.	.	tereti	teriti		sp
1	43	.	1330	7	[communi] desse	[id] esse (sum 1330)	desse	ok
1	50	1330	508	24	est [, vacuas]	ut [vacuas]	superest (supersum)	ok
1	66	10	.	23	tendere	tollere	tollere	ok
1	68	2	.	9	fana	fama	fama	ok
1	70	.	0	1	effringere	confringere 0	ecfringere [effringo]	NB
1	71	.	.	.	cupiret (cupio)	cuperet.		sp
1	74	.	.	.	omne	omnem		≈
1	77	.	.	.	qua nam	Quantum	quanam [quisnam] (5) sp	=?
1	84	.	.	.	Triviai	triviat		sp
1	102	.	.	.	quovis	quodvis		≈
1	104	.	.	.	possunt	possum.		≈
1	111	.	.	.	timendumst	timendum.	timendum	≈[±est]
1	121	104	.	21	eidem (104)	edens. (21)	edens (21)	ok
1	126	.	.	.	coeppise [et rerum]	Coepisset [rerum]		≈
1	130	.	8	104	tum	tunc		ok
1	141	4	.	5	sufferre	efferre	efferre	ok
1	158	.	508	1238	et	ut		ok
1	168	.	.	.	certa (83)	derta.		sp
1	176	.	1306	113	quia (113)	qui		ok
1	177	.	.	.	creatur	orcatu		sp
1	190	.	.	.	crescere [, resque]	Crescentesque	crescentesque	≈
1	207	.	.	.	possint	possent		≈
1	215	.	13	161	quique [in]	quicquid in (quisquis)	quicque ≈	ok
1	230	1	5	5	extentaque (1)	eternaque	externaque (5)	ok
1	240	.	.	.	nexu	nexus		≈
1	257	.	.	.	pingui	pinguis		≈
1	263	.	0	217	ex alio (217)	ex allo (allus,0)		NB
1	264	.	0	2	adiuta (2)	adluta (alluto,0)		NB
1	271	.	.	.	cautes (3)	cortus.	≠	sp
1	276	.	17	92	ventus (92)	pontus. (17)	ventus	ok
1	282	.	970	1361	quam	quem		ok
1	286	.	.	.	turbidus	turbibus		sp
1	289	.	.	.	ruitque [quidquid]	ruit qua [quidquid]	ruit qua	=?
1	294	0	.	8	rapideque (adv) ABSENT	rapidique (adj)	rapidique	ok
1	321	53	.	33	[praeclusit] spatium	[praecluset] speciem	speciem	ok
1	334	.	.	.	[different line]	Qua propter…vacanesque.		≠
1	347	.	.	.	licet	liceret		≈
1	349	.	228	2	flent (fleo)	fient (fio)		ok
1	352	.	0	9	radicibus (radix)	radicimus (radicio,0)		NB
1	357	.	16	228	[ulla] fieri [ratione]	[ulla] valerent [ratione] (valeo)		ok
1	366	.	153	114	at	Aut		ok
1	372	.	19	4	aiunt (aio)	alunt (alo)	aiunt	ok
1	383	.	0	4	initum	initium (0)		NB
1	384	.	2	8	concursu (n)	concurso (v)		ok
1	386	.	2	228	fiat (fio) (228)	flat (flo,2)		ok
1	389	.	.	.	quemque	quisque		≈
1	395	.	8	5	denserier (denseo)	condenserier (condenso)	denserier	ok
1	404	.	108	44	ferai (fera,44)	ferare.		ok
1	411	.	.	.	plano	ptano		sp
1	412	.	1	198	magnis	magnes.		≈
1	414	.	.	.	verear	urear		sp
1	438	.	.	.	transeire [Ernout transire]	transere		sp
1	442	.	.	.	possint	possunt		≈
1	449	.	.	.	cluent	civent		sp
1	449	.	.	.	coniuncta duabus	coniuncto duobus		≈/≈
1	451	0	.	1	perniciali 0	permitiali.	permitiali	ok
1	452	.	.	.	gregari	greagari.		sp
1	453	.	.	.	saxist	saxis		≈
1	453	.	.	.	aquai	aquae.		≈
1	454	.	.	.	[line missing]	Tactis … inani.		≠
1	455	.	.	.	divitiaeque (4)	diviaeque.		sp
1	458	.	.	.	eventa (evenio,pt)	evento		≈
1	467	.	.	.	fuerunt	fuerit.		≈
1	469	.	.	.	aliut per sest	aliunt terris		=?
1	473	.	.	.	formae	forma		≈
1	480	.	0	9	cluere	luere (luo, 0)		NB
1	484	.	1216	1191	quae	qua		ok
1	489	.	30	42	fulmen caelum	flumen caeli	fulmen caeli	ok/≈
1	490	.	162	120	[it] ac	[ut]ad		ok
1	490	41	.	508	it [ac] (eo,v)	ut [ad]	ut	ok
1	491	.	.	.	ferventia	ferventi		≈
1	492	104	.	413	tum (104)	Cum	cum	ok
1	500	.	.	.	constent	constet		≈
1	517	.	.	.	inane in rebu'queat	inane queat rerum		≠
1	520	.	.	.	sunt	est		≈
1	520	7	.	18	vacaret (vaco)	vocaret. (voco)	vocaret	ok
1	527	.	.	.	inane	inani.		≈
1	527	.	9	1	pleno	poena		ok
1	533	.	17	2	findi (findo)	fundi (fundo)		ok
1	542	.	.	.	renata (3)	ranta		sp
1	552	.	.	.	materiai (poetic)	materiae.		≈
1	553	.	2	1330	forent (sum, 1330)	fovent (foveo)	forent	ok
1	555	.	.	.	summa	summam		≈
1	562	.	.	.	refici (reficio)	refice		sp
1	562	.	103	374	videmus (374)	demus. (do)		ok
1	569	.	.	.	admixtum	Admixtumque		≈
1	578	62	953	161	quaedam	quae	quaeque	ok
1	580	.	.	.	clueant (9)	civeant		sp
1	584	.	.	.	generatim	generatin		sp
1	585	.	.	.	crescendi	Crescendis		≈
1	588	.	.	.	constent (consto)	constant.	constant	≈
1	588	.	1	11	commutatur (commuto)	comitatur (comitor)		ok
1	591	.	.	.	immutabili	Inmutabiles		≈
1	599	.	.	.	quia nam	quoniam		r
1	600	77	.	173	ullius	illius	illius	ok
1	606	.	.	.	explent	explet.		≈
1	608	.	77	93	nulla	ulla		ok
1	611	77	.	173	ullorum	illorum	illarum (≈illorum)	ok
1	613	.	51	138	iam	tam.		ok
1	619	.	1326	253	quid	quod		ok
1	626	.	.	.	constent	constant	constent	≈
1	628	4	.	215	ni	si	si	ok
1	631	274	.	93	multis	nullis	nullis	ok
1	634	.	1159	1191	quae	quas		ok
1	646	.	5	98	[ex] uno (unus) (98)	[Ex] uro (uro, -ere)		ok
1	649	.	.	.	habet	haberet.		≈
1	651	.	.	.	languidior	Langidior		sp
1	657	6	.	7	adesse (assum,6)	muse. (med. musa, musae,7)		ok
1	659	.	3	34	vera [viai] (adj)	ver (adv) [aula.]	vera viai	ok
1	659	.	0	52	vera viai	ver aula. (aula,0)	vera viai	NB
1	660	.	.	.	inani	inane.		≈
1	662	.	2	6	raptim (adv)	raptis. (raptio,n)		ok
1	665	.	1	217	alia	mia (adj)	alia	ok
1	666	.	.	.	coetu	coetus		≈
1	666	.	.	.	mutareque (49)	musareque		sp
1	668	.	20	34	[funditus] ardor	[funditur] arbor.	ardor	ok
1	670	.	0	16	mutatum (muto)	mutuatum (mutuor, 0)	mutatum	NB
1	674	.	2	2	vigescat (vigesco)	vivescat (vivesco)	vigescat	ok
1	680	4	4	15	decedere	descendere	discedere [discedo]	ok
1	683	.	.	.	crearent	crearet.		≈
1	690	.	.	.	ignem	iquenem		sp
1	694	.	.	.	labefactat (4)	laebefactat		sp
1	708	.	.	.	putarunt (73)	putantur.		≈
1	710	.	.	.	vertier (verto,old inf.)	verti (verto,p.inf.)		=
1	711	.	20	62	longe (adv)	longi		ok
1	716	.	.	.	Acragantinus	Agragantinus		sp
1	720	0	.	35	undans (undo)	undis. (unda) (35)	undis	ok
1	721	.	.	.	Italiae	Haeliae	Aeoliae (1)	sp
1	724	.	.	.	[ut] vomat (vomo) (2)	[ut]omniat		sp
1	725	.	.	.	flammai (poetic)	flammae		≈
1	739	.	0	2	profatur (profor) (2)	prosatur. (prosero,0)		ok
1	741	.	60	10	casu	causa.	casu	ok
1	744	.	.	.	fruges	frugis.		≈
1	747	.	.	.	faciunt	facient		≈
1	748	57	1306	84	quicquam	qui.	quire [queo]	ok

1	753 .		12	13	accedit [utei]	accidit [item]		ok
1	753	508 .		64	[accedit] utei (ut, uti)	[accidit] item	accedit item	ok
1	755	52 .		4	usque	utqui.	utqui	ok
1	758 .	.	.		habebis	habes.		≈
1	759 .		57	1	veneno (1)	vene.	veneno	ok
1	767 .	.	.		alternis	Aternis		sp
1	772 .		1238	508	ut	et		ok
1	774 .		117	37	animans (37)	animas (117)		ok
1	775 .	.	.		quicque in coetu	quisque in coetum		≈/≈
1	776 .	.	.		ostendet	ostendit		≈
1	777 .	.	.		???	et quodam cum	[as in Lachmann]	≠
1	777 .		15	41	manere	manare.	manere	ok
1	778 .	.	.		necessest (necesse+est)	oportet. (1)	oportet	≠
1	779 .	.	.		clandestinam	glandestinam		sp
1	780 .	.	.		emineat	Demineat		sp
1	781 .	.	.		creatur (creo)	creatas.		≈
1	784 .		160	37	imbrem	ignem		ok
1	785	59	160	37	gigni	igni	imbri [imber]	ok
1	785 .		900	177	a [terra]	in [terram]		ok
1	789 .		124	35	pacto (pactum,33,+pactus,2)	facto.		ok
1	806	1 .		17	ambusta [amburo]	arbusta	arbusta	ok
1	814 .	.	.		multa modis	multimodis		r
1	824 .		24	32	verbis	bellis.	verbis	ok
1	830	508 .		1238	ut	et	et	ok
1	834	413 .		1361	quom (=cum)	quam	quam	ok
1	835 .		150	349	e [ex]	de	e	ok
1	837 .	.	.		sanguenque	sanguemque		sp
1	843	349 .		104	ex parte	iden (=idem) parte	idem parte	ok
1	846 .		1326	1233	supra quos	suira quod		sp/ok
1	847 .	.	.		inbecilla (imbecillus,adj)	inbecilia		sp
1	852 .		17	9	effugiat	efficiat	effugiat	ok
1	853 .	.	.		† sanguin, os, aurem † (†=corrupt)	sanguis an os.		≠?
1	860 .	.	.		sanguen (=sanguin)	sanguin		=
1	861 .		55	21	sive	sine	sive	ok
1	861 .		0	542	corpore	core (coris, 0)		NB
1	862 .	.	.		esse et habere	esset habere		r
1	866 .	.	.		mixto (misceo)	mixta.	mixto	≈
1	866 .		1	30	sanguine	sanieque (sanies)		ok
1	874	542 .		16	[quae] corpora	[quae] lignis	lignis	ok
1	882 .		900	41	[cum] saxi (saxum)	[cum] in		ok
1	884 .		60	10	[]lapidi lapide] terimus (tero)	[in lapidem] tenemus (teneo)	terimus	ok
1	885 .	.	.		herbas	herbis		≈
1	886 .	.	.		laticis (latex)	latices		≈
1	890 .		900	120	inter	in	inter	ok
1	900 .	.	.		flammai (poetic)	flammae		=
1	906 .	.	.		conficerent	Conficeret		≈
1	909 .		17	9	contineantur	contingantur.	contineanteur	ok
1	912 .		349	1238	et	e		ok
1	918 .	.	.		hac	Haec		≈
1	919 .	.	.		tremulo	taemulo		sp
1	931 .		152	198	magnis	magis	magnis	ok
1	932 .		0	15	pergo	porgo. (=porrigo, 0)	pergo	NB
1	942 .		124	2	pacto	facto (facio)	pacto	ok
1	943 .	.	.		videtur	videt.		≈
1	954 .		395	3	necne sit (necne,3)	Nec sit	necne sit	≈/ok
1	957 .	.	.		immensum	imensum		sp
1	966 .	.	.		omnis	omnus.		sp
1	971 .		900	314	id	In		ok
1	981 .	.	.		fiat (fio)	fiet.		≈
1	984 .	.	.		spatium	scatium		sp
1	985 .	.	.		inclusum	inclusus		≈
1	987 .	.	.		confluxet	confluxit		≈
1	993 .	.	.		nullast (+est)	nullas		~
1	994 .	.	.		possint	possit.		≈
1	996	47 .		3	aeternaque	infernaque	infernaque	ok
1	1008 .	.	.		ipsa	Ipso		≈
1	1009 .	.	.		inani	inane.		≈
1	1017 .	.	.		coetu	coetum		≈
1	1019 .	.	.		creasset (97) (creo)	oreasset		sp
1	1023 .	.	.		quaeque…	quaeque… locarunt.		≈
1	1028 .	.	.		rerum	rebus		≈
1	1033 .		59	8	summissaque	summaque	submissaque	ok
1	1034 .	.	.		floreat	Floreant		≈
1	1037 .	.	.		amissa (amitto part.) (15)	ammissa		sp
1	1040 .	.	.		dissolui	Dissoluit		≈
1	1041 .	.	.		viaque	via.		=
1	1047 .	.	.		principiis (principium)	Principium	principiis	≈
1	1061 .	.	.		adsimuli	Et simili	simuli	r
1	1068 .	.	.		stolidis	stollidis		sp
1	1071 .		96	184	neque	denique	neque	ok
1	1073 .	.	.		alio	alia		≈
1	1074	70 .		900	[quod] inane (70)	[quod] in.	in	ok
1	1076 .		19	10	aeque	Aequis (aequus)		ok
1	1077 .	.	.		venerunt	vener.		≈?/sp
1	1082	2 .		29	vectae (veho)	victae. (vinco)	victae	ok
1	1091 .		316	26	[se] ibi (26)	sibi		ok
1	1105	3	1	1	penetralia	tonetralia	tonetrilia (NB: i)	ok
1	1106 .	.	.		omnia	omnis		≈
1	1114	2 .		4	perdoctus (perdoceo)	perductus (perduco)	perductus	ok
1	1114	32 .		87	scio	sic	sic	ok
2	12 .	.	.		labore (22)	plabore.		sp
2	15 .	.	.		periclis (pericles)	perictis		sp
2	17	1191	1306	4	cui	qui.	utqui	ok
2	18 .	.	.		menti	mente		≈
2	19 .	.	.		semotu'	semota (semoveo)		≈
2	27 .	.	.		fulgenti	fulget		≈
2	28 .	.	.		aurataque	iaquaeta		sp
2	28	13 .		25	tecta	templa.	templa	ok
2	39 .	.	.		super est	super es		=/≈
2	41 .		1330	5	fervere	Fuere (sum 1330)	fervere	ok
2	42	2 .		1	[magnisque] elephantis (!!)	[magnis] epicuri	epicuri	ok
2	43 .	.	.		validus [pariterque] (45)	itastuas [tariterque]		sp?
2	43 .	.	.		[validus] pariterque (29)	[itastuas] tariterque		sp
2	46 .		147	43	pectus	tempus	pectus	ok
2	52 .		3	3	purpureai (purpureus)	purpura. (purpura)		ok
2	54 .		0	22	laboret (laboro,22)	raboret. (rabo/rabio,0)		ok
2	62 .	.	.		materiai	materiae.		≈
2	68 .	.	.		quamque videmus	quamquidemus.		≠
2	73 .		4	8	augmine (8)	agmine (agmen)		ok
2	84 .	.	.		ferri [ferro] (108)	terri. (≠tero,terreo)		sp
2	86 .	.	.		conflixere (confligo,4)	conflexere		sp
2	86 .	.	.		ut	uti		=
2	88 .	.	.		tergo ibus	tergibus		=?
2	94 .	.	.		ostendi et	ostendit et		≈
2	95 .		274	93	nulla	multa	nulla	ok
2	99 .	.	.		brevibus (brevis,7)	brevius		sp
2	105 .	.	.		paucula (1)	Paucuia		sp
2	112 .	.	.		memoro	memoror		≈
2	112 .	.	.		simulacrum	simulacra		≈
2	134 .		8	4	conciliatu (n)	conciliata. (concilio,v)		ok
2	136 .	.	.		cientur [cieo] (27)	gientur.		sp
2	137 .		74	6	proporro	porro	proporo	ok
2	144 .	.	.		novo (v)	novor		≈
2	147 .		0	22	soleat (soleo)	solea (n,0)		NB
2	152 .	.	.		quasi (88)	quosi		sp
2	155 .		12	2	retrahuntur	trahuntur	retrahuntur	ok
2	158 .	.	.		remoratur (remoror)	remoravit.		≈
2	160 .		10	2	conixa (connitor)	conexa (conecto,pt)		ok
2	163 .	.	.		multiplexque (multiplex,2)	Multiplesque		sp
2	166 .	.	.		ut videant	Ut deant		sp
2	168 .		60	18	rentur [reor]	reddi. [reddo]	rentur	ok
2	181 .	.	.		[tanta stat] praedita (praedo,pt)	[quamquam] predita (medievalism)		=
2	184 .	.	.		illud quoque	quoque illud		vv
2	192 .		28	4	tigna (tignum)	signa (signum,n,signo,pt)	tigna	ok
2	193 .		1	8	subigenta (subigo)	subiecta (subicio)	subigente	ok
2	194	1330 .		349	est	e	e	ok
2	197 .	.	.		alte	altu.		sp
2	198	1 .		2	deiecta (deicio)	Derecta (dirigo)	derecta	ok
2	199 .	.	.		removit	removet		≈
2	200 .	.	.		exiliantque	exillantque.		sp
2	203 .	.	.		debent flamme quoque	quoque debent flamme		vvv

2	205	.	14	1330	quantum est	quantum inest	quantum in sest (sum)	ok
2	209	.	.	.	cadere in terram	caderem in terra		≈/≈
2	218	.	4	5	ferme	firme.	ferme	ok
2	219	0	.	2	[loci spatis] decellere	[locis spatio] depellere	depellere	ok
2	222	.	.	.	imbris	Imbres		≈
2	227	.	.	.	plagas	plag.		sp
2	229	.	0	10	avius (10)	Aulus (0)	auius (avius)	ok
2	233	.	.	.	aeris haut	Haeraut		r
2	234	.	.	.	exsuperata	exsuperate.		≈
2	240	.	.	.	poterunt	potuerunt		≈
2	250	11	.	517	praestet [praesto]	possit	possit	ok
2	251	.	.	.	motus	motu		≈
2	257	29	25	10	potestas	voluptas.	voluntas	ok
2	264	.	938	22	equorum	quorum. [qui 1]	equorum	ok
2	266	.	.	.	materiai (poetic)	materiae.		≈
2	268	.	10	2	conixa [connitor]	conexa [connecto]	conixa	ok
2	275	.	.	.	perspicuumst nobis	Perspicuum nobisst		vv w/ est
2	278	.	.	.	pellat (7)	Pallat		sp
2	279	.	.	.	rapi	rapit		≈
2	279	.	.	.	[in] pectore [nostro] (43)	iectore [no].		sp
2	279	.	1	95	[in pectore] nostro	[iectore] no. (1)		ok
2	281	.	3	28	copia	cona [conus]	copia	ok
2	283	.	.	.	residit	residia.		sp
2	289	.	628	80	ne mens	ne res	mens	ok
2	291	.	.	.	quasi [id cogatur] (88)	quaei cogatur (med. quae?)		sp?
2	294	.	.	.	fuit	fult		sp
2	301	.	.	.	vique	viquo		sp
2	305	.	.	.	in omne	in omnes.		≈
2	313	.	.	.	ipsa	ipsum.		≈
2	314	.	.	.	surpere (surripio)	asurpere	surpere	sp
2	318	.	1361	161	quamque [quisque]	quam	quamque	ok
2	320	.	.	.	satiati	satiat		≈
2	322	.	.	.	ut	uti		=
2	325	.	100	26	ibi	ubi	ibi	ok
2	337	.	.	.	constent	constat.		≈
2	342	.	.	.	parturiunt (parturio)	Praetere	Praeterea	sp
2	343	.	17	5	armenta	arbusta	armenta	ok
2	347	.	.	.	quidvis	quodvis		≈
2	354	.	1	34	calidum (34)	callidum (1)		ok
2	356	.	.	.	noscit (23)	Nonuit		
2	359	.	.	.	adsidueis, [et crebra revisit] (ass-)	adsittens [et revista crebra.] (as-si	adsistens (assisto,20)	sp
2	363	22	.	2	solitamque [soleo]	subitamque [subeo]	subitamque	ok
2	371	.	.	.	quodvis	quodviis	quoduis	sp
2	372	.	.	.	quique	Quidque		≈
2	381	29	.	170	tali	animi	animi	ok
2	382	.	.	.	ignis	igne.		≈
2	383	1330	.	33	fuat (sum, archaic,1330)	fluat (fluo,33)	fluat	ok
2	384	.	.	.	fulminis	fulmines		sp
2	387	.	.	.	ortu	ortus		≈
2	390	.	19	6	almus (6)	alimus (alo,19)	almus	ok
2	396	.	.	.	singula	singulta (singulto)		sp
2	403	.	.	.	[?no correspondence?]]	Nobilibus		≠
2	403	.	1	25	[iucunde] tangere (tango)	[iucudet] tacere (taceo)	tangere	ok
2	414	.	3	31	penetrare (v)	penetrale (n)		ok
2	421	.	34	3	diri (dirus)	di (deus)	diri	ok
2	422	.	.	.	videntur in 421?	videntur		≠?
2	423	.	0	1	levore (levor)	leviore (levior,0)		NB
2	423	.	153	36	haut = haud	Aut	haut	ok
2	427	.	.	.	unca	uncaque.		≈
2	428	.	.	.	angellis	angeliis		sp
2	429	.	.	.	titillare	Titiliare		sp
2	430	.	.	.	inulaeque (inula)	inviaeque		sp
2	430	.	1326	1233	quo	quod		≈
2	437	.	.	.	egrediens (egredior)	grediens		sp
2	438	370	.	153	atque	Aut	aut	ok
2	450	.	108	4	vociferantur	[voce] ferantur.	vociferantur	ok
2	451	.	.	.	e	ex		=
2	452	.	.	.	corpore	corpora		≈
2	453	.	1326	88	quasi	quod		ok
2	455	1	.	1	procursus =procurro part.	perculsis	perculsis	ok
2	458	.	.	.	omnibu’	omnia		≈
2	460	312	.	41	sese (sui)	saxa. (saxum)	saxa	ok
2	461	1	.	374	venenumst (veneno)	videmus. (video)	videmus	ok
2	462	.	.	.	sed rarum	sedatum (sedo)		r
2	465	60	.	77	habebis	debet.	debet	ok
2	467	.	349	1238	et [levibu’ sunt aliunde]	e [levibus atque rutundi]		ok
2	468	.	.	.	[retineri hamata] necessumst	[retinere hamat (sp)] necessu (sp).	[as Lachmann] necessum	sp
2	469	.	.	.	constent	constet.		≈
2	471	.	1326	1233	quo	quod		≈
2	476	.	.	.	primordia	mordia		sp
2	476	.	5	22	linquit (linquo)	Inquit (inquio)		ok
2	483	.	.	.	namque eadem unius	in cadem una (cadem≠eadem)		≠
2	486	.	.	.	paulo	paula		≈
2	488	.	.	.	transmutans	transmutas		≈
2	489	.	.	.	omnimodis	omnimodi		≈
2	495	.	.	.	augmen	augimen.		sp
2	497	.	5	108	semina (semen)	femina	semina	ok
2	499	.	.	.	probari	probare.		≈
2	501	25	13	1	tacta (tango part.)	tecta (tectum)	infecta	ok
2	502	.	.	.	ridenti [imitata]	rident [imbuta]		≈
2	502	8	.	3	[ridenti] imitata (imitor)	[rident] imbuta (imbuo)	imbuta	ok
2	503	.	.	.	novo	nova		≈
2	504	.	0	37	odor	udor		ok
2	514	.	34	22	finitis (finio, part.)	infinitis (n)	finitus	ok
2	515	.	2	96	denique (adv)	hiemisque (hiems, n)		ok
2	517	0	.	495	ambit (ambio)	Omnis	omnis	ok
2	518	.	.	.	interutraque	Inter utrasque		r
2	520	.	.	.	ancipiti	Ancipi		sp
2	521	.	.	.	hinc flammis	Hin flammps		sp/sp
2	521	.	.	.	infesta (infesto)	infessa		sp
2	529	3	.	17	Protinus	Versibus	versibus	ok
2	533	.	152	3	minis (minae)	magis	minus [parvum]	ok
2	535	.	.	.	genere	genera		≈
2	536	.	.	.	sicut	Sic uti		r
2	541	0	2	2	lubet	iubet.	quamlubet	ok
2	547	.	.	.	???	sumant oculi		≠
2	553	2	.	6	guberna	caverna	cavernas	ok
2	554	.	.	.	proram (prora)	prorem		sp
2	555	.	1	2	aplustra	plaustra.	aplustra	ok
2	560	.	34	22	finita (finio part.)	infinita	finita	ok
2	586	.	.	.	et quodcumque	Et quaecumque		≈
2	593	.	41	25	exiimus	Ex imis (eo)	eximiis	ok
2	605	.	.	.	molliri	moliri		sp
2	607	.	41	3	eximiis (eximius)	Ex imus (eo)		ok
2	613	.	.	.	orbem	orbes		≈
2	615	.	.	.	matris et ingrati	Matri et ingati		=/=/sp
2	615	.	.	.	sint inventi	inventi sint.		vv
2	623	.	.	.	numini’	numine		≈
2	626	.	0	10	iter [omne viarum]	ite [omnia virum.] (0)		NB
2	626	.	20	52	iter omne viarum (via)	ite omnia virum. (vir)	viarum	ok
2	628	.	.	.	catervas	caterva.		≈
2	630	.	.	.	forte quod armis	forte catervas.		=?
2	631	.	.	.	sanguinolenti	sanguine fleti.		r
2	632	26	.	17	nomine	numine	numine	ok
2	636	26	.	21	circum] puerum [pernice]	et in] numerum [pernice]	numerum	ok
2	637	.	.	.	armati	Armat (armo)		≈
2	656	.	.	.	constituit	Constituent		≈
2	663	.	1	5	sedantes (sedo)	sedentes (sedeo)	(in new 664)	ok
2	664	.	.	.	retinentque parentum	retinente parente		≈/≈
2	668	.	.	.	unam	una		≈
2	673	9	8	7	celant	traduntur.	condunt(7)	ok
2	674	.	.	.	ignem	igne		≈
2	680	.	22	3	parcat (parco)	parato. (paro)	(line repositioned)	ok
2	681	274	.	21	[privis] pluraque (multus)	[primis] pleraque	pleraque	ok
2	681	7	.	66	privis [pluraque] (privus)	primis [pleraque]	primis	ok
2	683	.	11	4	fucus	sucus	fucus	ok
2	684	.	11	4	fucus	Sucus	fucus	ok
2	685	7	.	66	privis	primis	primis	ok
2	692	.	93	274	multa	nulla	multa	ok
2	696	61	.	628	primordia verum	primordia rerum.	rerum	ok
2	706	.	.	.	omniparentis	omnia parentis		r

2	716	.		.		.		intus (adv)	inte		sp
2	719	.		.		.		omnia	omnis		≈
2	721	.			32		161	quamque	cumque	quamque	ok
2	730	.		.		.		dulci	dulce		≈
2	734	.		.		.		quo	que		sp
2	736	.		.		.		colore	colose (colos=color)		=
2	741	.			17		106	lumina	numina	lumina	ok
2	742	.			3		4	dispexere (dispicio)	Despexere (despicio)		ok
2	749	.			1238		900	in omnis	et omnis		ok
2	759	.		.		.		omne genus	omnigenus		r
2	760	.			95		47	propterea	Praeterea	propterea	ok
2	763	.			2		18	extemplo	exemplo	extemplo	ok
2	765	.		.		.		possint	possunt		≈
2	779	.		.		.		figura	figuras.		≈
2	780	.		.		.		ut	uti		=
2	781	.		.		.		aequore (aequor,n)	aequora		≈
2	783	.			36		65	colores	calores.	colores	ok
2	785		349	.			18	ex [his]	extra.	extra	ok
2	788	.		.		.		in licit et tribuamus	inlicitu tribuamus.		r
2	790	.		.		.		creantur	creatur		≈
2	791	.			314		349	ex	ea.		ok
2	791	.			71		395	nec	Ne	nec	ok
2	800	.		.		.		?recta aut	et aut		=?
2	800	.		.		.		refulgit	refulget		≈
2	803	.		.		.		rubra	rubro		≈
2	805		0	.			1	curalium	caeruleum	caeruleum	ok
2	806	.		.		.		larga	largo		≈
2	809	.		.		.		posse	posset		≈
2	814	.		.		.		sint	sunt		≈
2	814	.			198		152	magis	magnis	magis	ok
2	815	.		.		.		colores	colore		≈
2	821	.		.		.		omne genus	Omnigenus		r
2	829		9		24		6	aurea	aurum.	austrum (auster? 5+1)	ok
2	831		13	.			1	[distracta est] dispergitur	[distractum et] disperditur	disperditur	ok
2	831	.			1238		1330	[distracta] est [dispergitur]	[distractum] et [disperditur]		ok
2	840	.			71		395	nec	Ne	nec	ok
2	841	.			13		11	notare (noto)	notaque. (n or v)		ok
2	842	.		.		.		spoliata	spoliita		sp
2	845	.		.		.		ieiuna (2)	etuna		sp
2	846	.		.		.		proprio	proprium		≈
2	850	.			1233		1326	quo	Quod		ok
2	853	.		.		.		contractans	contractas		≈
2	860	.		.		.		molli	Mollia		≈
2	867	.		.		.		constare (105)	consistare [≠consistere, 33)		sp
2	867	.			1		4	refutant	reputant.	refutant	ok
2	872	.			0		3	nacta (3)	inacta (inigo, 0)		NB
2	875	.		.		.		frondes	in frondes		≈
2	882	.			1238		900	in [ignis]	et [ignis]		ok
2	891	.			4		8	decebit (decet, decetere)	decedit.	decebit	ok
2	891	.			0		628	rebus	fedus (=haedus,0		NB
2	893	.		.		.		sensile [et extemplo] (adj)	Sensilia [etemplo]		sp
2	893	.		.		.		sensile et extemplo	Sensilia etemplo		sp
2	902		108	.			4	[ea,] seminibus	[ex] sensilibus	sensilibus	ok
2	903	.		.		.		suetis	sueti.		≈
2	904		138	.			412	[mollia] iam	[Mollia] cum	cum	ok
2	905		52	.			32	cuncta	cumque	cumque	ok
2	909	.		.		.		simili	similis		≈
2	910	.			153		114	at	Aut	at	ok
2	911	.		.		.		alio	alios		≈
2	911	.			6		5	respicit	respuit	respicit	ok
2	918	.		.		.		animalibus sint mortalibus	animalibus mortalibus		≈//≈
2	919	.		.		.		at coetu	arbeoretu		r
2	921	.		.		.		nequeunt	nequeant		≈
2	928	.		.		.		effervere	offervere		sp
2	928		0	.			19	alituum (alitus,n)	alitum (alo)	alitum	ok
2	929	.			1200		413	[intempestivos] quom (=cum)	[Intempestivus] quam	quom	ok
2	932	.			314		349	ex	ea		ok
2	933		3	.			8	protinus [extent]	proditum [extra.]	proditur	ok
2	934	.		.		.		huic	Icuic		sp
2	938	.		.		.		ipsam	lesam		sp
2	940		32	.			265	[terris] aetheraque ?	terraque	terraque	ok
2	942	.		.		.		omnituentes	omne tuentes		r
2	943		20	.			24	[animantum] concuterentur	[animantem quoque] tuentur	tuentur	ok
2	951	.			3		8	caulas	cavias	caulas	ok
2	953	.			177		127	ac	a	ac	ok
2	954	.		.		.		[oblato] acriter (5)	[oblata] agriter		sp
2	961	.			11		11	possint conlecta (colligo, 10+1)	possint coniecta (conicio)	conlecta	ok
2	963		47	.			95	propterea	Praeterea	praeterea	ok
2	966	.		.		.		inque	In quo		r,sp?
2	975		124	.			29	factumst [?]	auctumst.	auctumst	ok
2	977	.		.		.		spargunt (9)	pargunt		sp
2	982	.		.		.		alia ex	ali ex		≈/=
2	985	.		.		.		delira (delirus, 3)	detira		sp
2	986		124	.			29	factus	auctus	auctus	ok
2	998	.			7		5	adepta (adipiscor,5)	adempta (adimo,7)		sp
2	1000	.		.		.		missumst (+est)	missus		≈
2	1002		46	.			628	ut materiai	ut res	res	ok
2	1004	.		.		.		[coniungitur et] fit [ut omnes]	[coniungit et] efficit [omnes.]	efficit	r
2	1007		628		314		104	rerum	earum	eadem (idem)	ok
2	1011		52	.			60	cunctis	summis (superus)	summis	ok
2	1016	.		.		.		[different line]	Significant eum …amantis.		≠
2	1017	.		.		.		sunt	sint		≈
2	1017	.			25		208	pars	par	pars	ok
2	1020	.		.		.		[different line]	Intervalla… plagaes.		≠
2	1023	.		.		.		adhibe veram	adhibureram		r
2	1024	.			4		5	vehementer (adv)	vehementes		ok
2	1025	.			14		12	accidere	Accedere	accidere	ok
2	1029	.		.		.		[mittant] mirarier (miror,14)	[minuant] miraliter		sp
2	1029		63	.			8	mittant [mirarier]	minuant [miraliter]	minuant	ok
2	1030		18	.			82	percipito (percipio part.?)	Principio	principio	ok
2	1031	.		.		.		cohibet	cohibent		≈
2	1033		26	.			1330	extent (exsto)	essent. (sum)	essent	ok
2	1047	.			23		3	iactus [liber]	tactus [libero]	iactus	ok
2	1047	.			0		10	[iactus] liber (10)	[tactus] libero (0)		
2	1049	.			24		12	supra	superque	supra	ok
2	1052	.		.		.		[veri] simile	[verist] mile (=mille)		r
2	1061	.		.		.		coluerunt (L-mannism?]	colerunt (colo)		≈?
2	1061		2	.			15	convecta	coniecta (conicio/coniectus)	coniecta	ok
2	1062	.			37		11	exordia	ex ordine	exordia	ok
2	1070		349	.			1238	ex	et	et	ok
2	1072		1275	.			198	quis	Vis	vis	ok
2	1073		84	.			114	quaeque queat (queo)	quaeque at	queat	ok
2	1078	.		.		.		gignatur	gignantur		≈
2	1079	.		.		.		aliquoiu' siet	aliquoivis		r
2	1080		3		3		2	inclute [, Memmi] (inclytus)	indice [mente] (indico)	inice	ok
2	1080		10	.			80	[inclute,] Memmi	[indice} mente	mente	ok
2	1081	.		.		.		invenies	Invenisse		≈
2	1082	.			5		1	genitam (genitus -a -um)	geminam (gemitus -a -um)	genitam	ok
2	1089		1330		314		411	quod est	quod his	hic	ok
2	1094	.			1		123	vitamque (123)	ultamque (ulciscor,1)		sp
2	1097	.		.		.		convertere	converteret		≈
2	1102	.			4		3	recedens	decedens	recedens	ok
2	1110	.		.		.		appareret (appareo)	Appariret		sp
2	1115		108	.			33	aeraque	aetheraque.	aetheraque	ok
2	1116	.		.		.		extremam	extremum		≈
2	1117	.		.		.		creatrix (3)	cracreatrix		sp?
2	1122	.		.		.		hilaro (1)	hilar		sp
2	1124	.			1		542	corpora	cora (also=caurus, pl.)		ok
2	1129	.		.		.		debent	debet.		≈
2	1135	.		.		.		ab se	a se		=
2	1136	.			3		16	diditur	deditur	diditur	ok
2	1138	.		.		.		queat	queant		≈
2	1145	.		.		.		putrisque	putris		≈
2	1148		71	.			395	ne	Nec	nec	ok
2	1150	.			17		2	effetaque	effectaque (efficio part.)	effetaque	ok
2	1150	.			124		18	fracta (frango)	facta (facio)	fracta	ok
2	1153	.		.		.		[opinor, enim] mortalia	[opinore] immortalia	mortalia	r
2	1160	.			1216		1191	quae	Qua		ok
2	1165		41	.			198	manuum	magnum	magnum	ok
2	1168	.		.		.		=? -fatigat in 1169]	fatigat.		=?
2	1169	.			26		5	momen [, caeculumque]	nomen [saeclumque]	momen	ok

2	1169		0	.		46	[momen.] caeculumque	[nomen] saeclumque	saeclumque	ok
2	1170	.		.	.		pietate	pletate		sp
2	1172	.		.	.		viritim (1)	diritim.		sp
2	1174	.			0	1	capulum (1)	scopulum (0)		NB
3	1	.			0	349	E	O (0)		NB
3	7	.		.	.		cycnis	Cycniis		sp
3	11	.			0	5	libant (libo,0)	limant. (limo,0)	libant	NB
3	15	.		.	.		coorta	coortam.		≈
3	21	.		.	.		semperque	semper		≈
3	22	.		.	.		rident	ridet.		≈
3	28	.			100	26	ibi	ubi	ibi	ok
3	29	.			34	236	[sic] natura (236)	signatura (signo pt.,34)		ok
3	33	.			3	47	aeterno	alterno	aeterno	ok
3	39	.		.	.		suffundens	suffundans		sp
3	47	.		.	.		causa	causam		≈
3	53	.		.	.		inferias	Inferia		≈
3	58		14	.		1	eiciuntur	Eliciunter	eliciuntur	ok
3	58	.			15	41	manet res	manare.		ok
3	62	.		.	.		labore (22)	tabore.		sp
3	66	.		.	.		videntur	videtur.		≈
3	72	.		.	.		fratris	fratres.		≈
3	78	.			5	1	statuarum	statum	statuarum	ok
3	81	.		.	.		coniscant	coniciscant		sp
3	84	.		.	.		pietatem	pietate		≈
3	84		5		7	1	fraude (5)	suadet. (suadeo)	suase (suasis)	ok
3	94	.			970	1361	quam	quem		ok
3	95	.			18	26	locatum (loco, part., 26)	vocatum (voco)	locatum	ok
3	100	.		.	.		faciat	taciat		sp
3	106	.			2	2	aegret (2)	aegrum. (2)		ok
3	108	.			100	508	uti	ubi	uti	ok
3	118	.			12	52	senteire (sentire)	interire (interiere)	sentire	ok
3	132	.		.	.		alto	altu		sp
3	151	.		.	.		novitate cietur	novitatficietur.		r
3	154	.			10	67	ita [palloremque] (67)	itaque [pallorem] (10)		ok
3	165	.		.	.		fieri	fleri		sp
3	170	.		.	.		offendit	offendis		≈
3	170	.			33	18	teli (18)	leti.	teli	ok
3	172	.		.	.		insequitur	insequetur		≈
3	173	.		.	.		mentis	mentes		≈
3	173		1	.		14	suppus	Suavis	suavis	ok
3	176	.			542	11	corporeus (adj)	Corporis (n)		ok
3	183	.			215	316	sibi	si		ok
3	198		4	.		1	[conlectum] spiritus	[coniectum] spicarumque.	spicarumque	ok
3	198	.			15	2	conlectum [spiritus]	coniectum [spicarumque.]	conlectum	ok
3	210	.			316	215	si	se		ok
3	222	.		.	.		unguentei (poetic i ?)	unguente		≈?
3	227	.		.	.		rei	rerum		≈
3	234	.		.	.		[cui mixtus non] siet [aer] (siem=su	[cui non] sit [mixtus et aer]		≈
3	236	.		.	.		moveri	mqueri.		sp
3	239		970	.		80	[recipit] quem	[recepit] mens	mens	ok
3	240	.		.	.		vis menti'	que mente		sp,≠/≈
3	243	.		.	.		tenvis	tenuis		=
3	244		1238	.		349	et parvis	e parvis	e	ok
3	249	.		.	.		sanguis, tum	tum sanguis		vv
3	249	.		.	.		persentiscunt	persentisiunt		sp
3	255	.		.	.		caulas	culvias		sp
3	257	.		.	.		valentes (pt)	valemus.		≈
3	272	.		.	.		sensifer	Sensiferer		sp
3	288	.			138	27	etenim	etiam	etenim	ok
3	289	.		.	.		fervescit	fervescet		sp
3	289	.		.	.		acribus (adj)	acrius (comp adj)		≈
3	290		314	.		1238	ea	et	et	ok
3	291	.		.	.		concitat	inconcitat (in-)		sp
3	293	.		.	.		pectore	Pertore		sp
3	293	.		.	.		qui fit	fit qui		vv
3	303	.			8	10	nimis (10)	minus (minor,8)		ok
3	304	.			2	5	[fumida.] suffundens	[Fumidas] effundens	suffendens	ok
3	305	.			44	4	pavoris	vaporis.	pavoris	ok
3	306		2	.		5	[interutraque] secus	[Inter utrasque] sitas (sino,pt)	sitast	ok
3	309	.		.	.		naturae	Natura		≈
3	319	.			2	4	[videor] firmare	[video] formare (formo)		ok
3	321	.		.	.		necqueat	niqueat		sp
3	321	.			28	161	nobis	noctis.	nobis	ok
3	330	.			153	36	haut	aut	haut	ok
3	332	.		.	.		fiunt consorti	consorti fiunt		vv
3	332	.		.	.		vita	vitae.		≈
3	335	.		.	.		eas	eos		≈
3	346	.		.	.		reposta	reposto		≈
3	357	.		.	.		perdit	Perdi		≈
3	358		93	.		274	nullaque	Multaque	multaque	ok
3	358	.			1169	413	perdit,] quom [expellitur aevo	perditum expellitur aevo [quam.	cum	ok
3	361		3	.		8	despicerest (despicio, 3)	Difficilest (comp)	difficilest	ok
3	361		76	.		25	dicat	ducat	ducat	ok
3	365		113	.		1216	quia	qua	qua	ok
3	372	.			66	7	privis	primis.	privis	ok
3	374	.		.	.		elementa minora animai	animae elementa minora.		vvv
3	378		7	.		66	priva	prima	prima	ok
3	380		7	.		66	priva	prima	prima	ok
3	383	.		.	.		[aranei] tenvia	[arani] tenulla		sp
3	391	.		.	.		ciendum	ciendo.		≈
3	394	.			23	1361	Et quam	Et quantis (quantus)	et quam	ok
3	399	.			1	7	exiguam (exiguus)	exigiam (exigio)		ok
3	400	.			349	1238	et	e		ok
3	403	.			1	45	circum	cretum (creto,pt)		ok
3	404	.		.	.		remota	remot.		sp
3	405		8	.		14	aerias	aetherias	aetherias	ok
3	412	.		.	.		[different line]	Id quoque … eorum.		≠
3	415	.		.	.		[different line]	Incolumis … orbis.		≠
3	417	.		.	.		mortalis	mortalibus		≈
3	420		22	.		123	cura	vita.	vita	ok
3	421	.		.	.		uni	uno		≈
3	421	.			0	26	nomen	nome (nome,0)	nomine	NB
3	428		138		180	108	iam	nam	nam	ok
3	429	.		.	.		praestat	Prestata		≈
3	430	.		.	.		[different line]	Quippe … moventur.		≠
3	431	.			58	21	alte (n)	alta. (alto,v)		ok
3	431		1330	.		900	est, somnis	in somnis	in	ok
3	432	.		.	.		vaporem	vapore		≈
3	433	.		.	.		[different line]	Nam procul … geruntur.		≠
3	437	.		.	.		crede	Credeo		sp
3	438	.		.	.		ocius	Opius		sp
3	441		1361	.		413	quam	Cum	cum	sp
3	444	.		.	.		[is] cohibessit	[in] cohibescit (?cohibesco?)	incohibens	sp
3	444		41		413	1	is (41)	in (413)	incohibens (1)	
3	444		314		900	152	is [cohibessit]	in [cohibescit]	magis [incohibens] NB	sp
3	449	.		.	.		robustis	rubustis (rubigo=robigo)		=
3	450	.			0	1	auctior (aucto/auctio,v)	auctor (n,0)	auctior	NB
3	456	.			46	108	aeris	acris	aeris	ok
3	458	.		.	.		fatisci (fatisco)	faetis.		sp
3	462	.		.	.		participem	particidem		sp
3	470	.		.	.		fateare (fareor)	fatere		sp
3	472	.		.	.		dolor	polor		sp
3	475	.		.	.		[different line]	Et pariter … inani		≠
3	479	.		.	.		vacillanti	vaciliaanti		sp
3	482	.		.	.		cur ea	Curba		=?
3	492	.			1216	113	quia	qua		ok
3	493	.		.	.		spumat	spumans in		≈
3	497	.			1	14	eiciuntur	Eliciuntur	eicitur	ok
3	522	.			0	50	docui	deocui (deocco,0)		NB
3	523	.		.	.		rationi	rationis		≈
3	525	.			0	1	refutatu (n)	refutator (n,0)		NB
3	531		52		381	1	usque [adeo]	atque [animo]	atqui	ok
3	531		50		170	117	[usque] adeo	[atque] animo (animus)	animae NB	ok
3	535		5	.		5	deducere (5)	diducere	diducere	ok
3	544	.			0	13	dispersa (dispergo,v)	disperse (adv+dispersus,0)		NB
3	545	.		.	.		obbrutescat	obruscat		sp
3	551		153	.		381	aut	atque	atque	ok
3	553		1		0	3	lincuntur	linguntur (lingo,0)	liquontur NB	ok
3	555	.		.	.		homine	hominem		≈
3	555	.		.	.		vas	vasse		sp
3	564	.		.	.		ipse oculus	oculus ipse		vv

3	566	.			36		1	mixtim (=mistim.adv.1))	mixti (misceo, 36)		ok
3	571	.		.		.		moveri	movere.		≈
3	573	.			117		38	animans [erit]	animam [serit] (animas)		ok
3	573	.			1		1330	[animans] erit (sum)	[animam] serit (sero)		ok
3	574	.		.		.		in se animam	Sese anima		≈/≈
3	576	.			1191		43	quare	Quae		ok
3	578	.		.		.		fateare	fatiare		sp
3	580	.		.		.		nequeat (nequeo)	nequea		sp
3	582	.			314		349	ex imo	ea imo		ok
3	583	.			314		198	[animae] vis	[anima] eius	vis	ok
3	586	.		.		.		manante animaque	manant animaeque (mano)		≈
3	594	.		.		.		[unclear correspondence]	corpore omnia membra.		≠
3	596	.		.		.		exsangui	easangui		sp
3	597	.		.		.		peribetur	peribet.		≈
3	612	.		.		.		iNmortalis	iMmortales		=
3	617	.			495		29	regionibu’	omnibus	regionibus	ok
3	618	.			24		60	reddita (reddo)	redita (redeo)		ok
3	620	.		.		.		ita	ta		sp
3	620	.		.		.		perfectis	pertotis		sp
3	623	.		.		.		[solistast neque in] igni	[solita neque] insigni	igni	r
3	624	.		.		.		animaist	animaest.		≈
3	624	.			68		21	iNmortalis	mortalis	inmortalis	ok
3	626	.		.		.		auctam	auctum.		≈
3	628	.			7		16	vagari	vacare.	vagari	ok
3	632	.		.		.		animae	anima		≈
3	633	.			9		36	haud	Auditum		ok
3	644	.			162		177	ab [artubus]	ad [artubus]	ab	ok
3	645	.		.		.		decidit	Dicidit		sp
3	647		15	.			38	semel	simul	simul	ok
3	650	.		.		.		rotas	rote		sp
3	651	.		.		.		instat	istat.		sp
3	657		3	.			8	micanti	minanti.	minanti	ok
3	658	.		.		.		serpentum] cauda, [e procero (n)	Serpentis] caude [procero (v)		r
3	658		9	.			5	utrimque	utrumque	utrumque	ok
3	662	.		.		.		retro	reretro		sp
3	663	.		.		.		dolorem	dolore.		≈
3	674	.		.		.		operest animi	opere animist		vv est
3	676	.		.		.		a	ab		=
3	676	.		.		.		longier (poetic?)	longius (longum.comp)		≈
3	680	.		.		.		solitast animi	solita animist		vv est
3	685	.		.		.		arceat	afluat (≠word)	affluat	sp
3	691	.		.		.		per venas viscera	viscera per venas		vv
3	693	.		.		.		aquai	aque.		=
3	693		7	.			40	morsus	Morbus	morbus	ok
3	694	.		.		.		[expressus] subiens	[oppressus] subitis	subitis	≈
3	694		17	.			8	expressus [subiens]	oppressus [subitis]	oppressus	ok
3	702	.		.		.		disperitus [enim] (6)	Dispiritur [ergo]	disperitur	sp
3	702	.			41		181	[disperitus] enim	[Dispiritur] ergo	enim	ok
3	705	.		.		.		quamvis	quamvis est		=
3	710	.			8		104	tum	tunc	tum	ok
3	718	.			1238		508	ut	Et		
3	719	.		.		.		viscere	vicere		sp
3	723	.			215		900	privas in	priva si		≈/
3	732	.		.		.		alguque	algoque		sp
3	734	.		.		.		contagibu’	contage		≈
3	736	.		.		.		qua	que (med. quae)	cui	≈
3	738		138		1306		4	ut iam	ut quicum (≈qui)	utqui NB	ok
3	740	.		.		.		consensus	consensu		≈
3	743	.		.		.		[different line]	A patribus … artus.		≠
3	760	.		.		.		corpora	corpore		≈
3	760	.			87		6	sin	Sic	sin	ok
3	764		3		20		77	pullus	paulus	ulla NB	ok
3	775	.			68		21	immortali	[iam] mortali	immortali	ok
3	784		16	.			58	salso (1+15)	in alto. (12+46)	in alto	ok
3	785	.		.		.		nubes	Nube		≈
3	789		0	.			20	longiter (adv)	longius (comp adj)	longius	ok
3	790		253	.			1326	quid	Quod		ok
3	798		0		0	.		periisse (perior)	perisse (peror,0)	=?	NB
3	800	.		.		.		mortale	mortalem		≈
3	804	.		.		.		perenni	peranni.		sp
3	805	.			1		15	saevas	salvas (1)	saevus	ok
3	820		0	.			123	letalibus	vitalibus	vitalibus	ok
3	824	.		.		.		morbis	morbist		~
3	824	.		.		.		aegret	aegrit.		sp
3	826	.		.		.		macerat	Maceret		≈
3	829	.		.		.		adde	Addi		≈
3	835	.			68		33	oris	auris.	oris	ok
3	844	.		.		.		distractast	Distractas		~
3	851		1		3		1	retinentia [nostri] (1)	repentia [nostris] (repo)	repetentia NB	ok
3	853	.			5		7	adficit (afficio)	adfigit (affigo)	adficit	ok
3	853	.			14		1330	fuimus	fumus (14)		ok
3	856	.		.		.		multimodis	Multimodi		≈
3	861	.		.		.		misere	miserest		~
3	863	.			7		11	probet	prohibe.	probet	ok
3	863	.			1		57	mors	mox		ok
3	868	.		.		.		[ante] ullo	annulo anulo		=?
3	871	.			73		1	putescat	putes	putescat	ok
3	873	.			1		281	non	no		ok
3	877	.		.		.		radicitus (adv)	radicitius		?sp/?~
3	880	.			30		6	lacerent	iacerent	laceret	ok
3	886	.			1191		1306	qui	Cui		≈
3	887	.			29		2	dolere	dolore.		ok
3	890	.		.		.		torrescere	torreresecere		sp
3	893	.			6		3	obtritum	obrutum	obtritum	ok
3	894	.		.		.		iam	Am		sp
3	897	.		.		.		factis	facti		≈
3	902	.			1233		1326	quod	Quo		ok
3	906	.		.		.		cinefactum	[cinem] factum		r
3	908	.			1238		349	e	et		ok
3	910	.			312		215	si	se		ok
3	914	.			20		8	fructus	fluctus	fructus	ok
3	917	.		.		.		torres	torret.		≈
3	919	.		.		.		requirit	requiret.		≈
3	922	.			5		7	adficit (afficio)	adigit	adficit	ok
3	941	.		.		.		offensust	offensost		≈
3	943	.			23		124	facis	iacis	facis	ok
3	945	.		.		.		placeat	placet		≈
3	947	.		.		.		languent	languente		sp
3	947	.		.		.		restant	restat.		≈
3	948	.		.		.		pergas	perges		≈
3	950	.			215		52	nisi	si		ok
3	954	.		.		.		balatro (1)	baratre		sp
3	958	.			0		1	inperfecta (adj)	Inperfecte (adv,0)	inperfecta	NB
3	960	.			6		15	discedere (discedo)	discere (disco)		ok
3	962	.		.		.		[age dum] dignis	[agendum] magnis	agedum iam aliis =?	r
3	964	.		.		.		cedit	Cedi		≈
3	966	.		.		.		barathrum	baratrum		sp
3	985	.			253		1326	quod	quid		≈
3	988	.			6		4	dispessis	dispersis	dispessis	ok
3	992	.		.		.		est	es		≈
3	994	.		.		.		cuppedine	curpedine		sp
3	997	.		.		.		tristisque	tristique		≈
3	999	.		.		.		sufferre laborem	laborem sufferre		vv
3	1005		29	.			45	victum	circum.	circum	ok
3	1009	.			66		2	congerere	cogere	congerere	ok
3	1010	.			77		93	nulla	ulla	nulla	ok
3	1011	.		.		.		furiae (1)	funae		sp
3	1013		253	.			1306	quid	Qui		ok
3	1014	.		.		.		poenarum	paenarum		sp
3	1015	.		.		.		luella	luela		=
3	1016	.			314		9	deorsum (9)	eorum.		ok
3	1017	.		.		.		lammina	iammina		sp
3	1019		0	.			4	terretque	torretque (4)	torretque	ok
3	1032	.			0		1	insultans (insulto)	insuitans (insuo,0)		NB
3	1033	.			20		22	fudit (fundo,5+17)	fugit.	fudit	ok
3	1038	.			23		6	potitus (potior part.)	potius (potis comp)		ok
3	1042		41	.			16	iit (eo)	obiit	obit	ok
3	1044	.			8		14	aetherius	aerius	aetherius	ok
3	1050	.			1326		253	quid	quod		≈
3	1052	.		.		.		animi	animo		≈
3	1061	.		.		.		quem pertaesumst	per quem		=?

3	1063	.	.	.	praecipitantur	praecipiter		sp
3	1064	.	.	.	instans	instas.		≈
3	1068	413	.	970	quom=cum	quem	quem	ok
3	1069	.	.	.	ingratis	ingratius (comp?)		~
3	1073	.	.	.	temporis aeterni	Aeterni temporis		vv
3	1075	.	.	.	manenda	manendo		≈
3	1078	.	0	8	[certa] quidem	[Certe] equidem (adv,0)		NB
3	1084	.	.	.	hiantis	hientis		sp
3	1085	.	.	.	fortunam	fortuna		≈
3	1088	.	0	4	delibare	deliberare (delibero,0)		NB
3	1089	.	.	.	possimus	possumus		≈
4	8	.	2	5	pango	pando.	pango	ok
4	14	.	1238	508	ut	Et		ok
4	17	.	.	.	pacto	atacto		sp
4	21	.	.	.	Pierio (name)	plerio		sp
4	41	.	.	.	quoique (=cuique)	quaeque (quisque)		≈
4	42	.	9	3	effigias (effigio)	effugias (effugio)		ok
4	43	.	2	542	corpore [rerum]	cortice [eorum.]	corpore	ok
4	43	.	314	628	[corpore] rerum	[cortice] eorum.	rerum	ok
4	51	.	1306	1191	quae	Qui		ok
4	53	.	.	.	cluet	civet		sp
4	54	.	.	.	mittunt	mittuntur		≈
4	63	.	.	.	debet	debe		≈
4	68	.	314	104	eodem	eorum	eodem	ok
4	72	.	.	.	largiri	iergeri		sp
4	77	.	20	2	flutant	fluctus.		ok
4	79	.	.	.	scaenai	Scaenal		sp
4	79	6	.	1	pulchram [variumque decorum]	patrum [matrumque deorum.]	patrum	ok
4	79	63	.	9	[pulchram] variumque [decorum]	[patrum] matrumque [deorum.]	matrumque	ok
4	79	1	.	34	[pulchram variumque] decorum	[patrum matrumque] deorum.	deorum	ok
4	81	.	.	.	angusta	inclaustra (in+claustra)		?
4	90	.	.	.	aliae	alia		≈
4	92	1	.	26	torte (1)	ortae. (orior, 26)	ortae	ok
4	101	.	349	2	excita	Ex		ok
4	101	.	314	628	rerum	eorum.	rerum	ok
4	104	.	1	42	similesque	milesque. (=mille)		ok
4	118	.	.	.	horum	Harum		≈
4	142	.	4	33	oras	horas.	oras	ok
4	143	.	39	3	genantur	gerantur.	genantur	ok
4	150	.	.	.	opposta (oppono)	opposita. (oppono)	opposta	≈
4	152	.	.	.	potis	possunt		≈
4	159	.	39	3	genuntur	geruntur.	genuntur	ok
4	175	.	.	.	rationem	ratione		≈
4	178	.	108	10	teratur	feratur.	teratur	ok
4	179	5	.	17	momine [tendat]	numine [tendit.]	numine	ok/≈
4	203	45	.	150	circumque	caelumque	caelumque	ok
4	210	.	.	.	motu	motum		≈
4	213	.	.	.	mundo	mundi.		≈
4	216	63	.	27	mitti ?	mira (adj)	mira	ok
4	218	.	.	.	fluunt	fluant		≈
4	220	.	.	.	moerorum	meororum		sp
4	229	.	.	.	[different line]	Cernere … sonare.		≠
4	235	.	.	.	commovet in	Commovet [et] in		~
4	237	1216	113	27	qua	quia	quapropter	ok
4	240	.	4	16	didita	dedita	didita	ok
4	245	66	.	9	cogit	curat.	curat	ok
4	246	.	.	.	protrudit	protudit.		sp
4	249	.	0	3	perterget (pertergeo)	perteget (pertego,0)		NB
4	253	.	.	.	remota	revota		sp
4	261	.	66	7	privam	primam	privam	ok
4	267	.	.	.	ipsam	ipsa		≈
4	270	.	10	3	semota	remota	semota	ok
4	271	.	.	.	? quod … ?	que vere		=?
4	277	.	0	3	perterget	perteget (0)		NB
4	278	.	.	.	?…?	que verre		?…?
4	283	.	.	.	speculum	inspeculum		sp
4	284	2	314	104	iterum	[in] eum	idem	ok
4	304	.	.	.	acer adurit	tacer ardurit.		sp/sp
4	309	.	.	.	multa	mault		sp
4	313	.	13	3	propior (3)	proprior (comp., proprius)	propior	≈
4	317	.	77	173	illius	ullius	illius	ok
4	323	.	.	.	elisam	lisam		sp
4	324	67	.	508	fiet ita	Fiet ut	ut	ok
4	328	.	.	.	latebunt	latebit		≈
4	334	.	.	.	convertitur	convertit		≈
4	341	.	.	.	flexa (flecto)	Flexea		sp
4	351	.	.	.	obsiditque	Obsidit quia		≠
4	352	.	0	11	coniecta (conicio,11) movere	contecta (contigo,0) moveri.	coniecta	ok
4	355	.	.	.	optusus (poetic~optimus?)	optutus		?sp?
4	357	.	.	.	plaga	Plagas		≈
4	361	.	.	.	tornum (1)	turnum		sp
4	361	.	.	.	tuamur	tuantur.		≈
4	368	.	.	.	aliut	aliui		sp
4	378	.	0	2	abluit (2)	adluit (alluo=adluo,0)		NB
4	395	.	.	.	videntur	videtur.		≈
4	397	.	.	.	exstant [usque]	Extentisque	extentisque	≈
4	406	.	100	126	tibi (tu, 126)	ubi		ok
4	414	.	4	3	conlectus (conligo part.)	coniectus	conlectus	ok
4	417	.	217	46	altus	alius	altus	ok
4	418	4	.	3	dispicere	despicere	despicere	ok
4	419	.	.	.	?	Corpora mirande		?=?
4	419	.	.	.	caeli	caelo.		≈
4	421	.	4	3	despeximus	dispeximus	despeximus	ok
4	423	.	30	42	flumen	fulmen	flumen	ok
4	429	.	.	.	coni (conus, 3)	cogni.		sp
4	436	.	177	114	at	A	at	ok
4	437	.	.	.	undae	undas.		≈
4	437	.	124	18	fractis (frango)	factas	fractis	ok
4	440	.	.	.	liquorem	liquore		≈
4	446	.	.	.	ac vera ratione	aque ratione		?=
4	448	.	.	.	uti	ut		=
4	453	.	.	.	suavi devinxit	sua videt vinxit		?=
4	456	.	.	.	videmur	videatur		≈
4	460	.	49	28	noctis	montis.	noctis	ok
4	462	.	.	.	miracli (med. contraction?)	mirande (miror)	mirande	sp?
4	471	.	.	.	mittam	mituam		sp
4	472	.	.	.	sua	suo		≈
4	475	.	.	.	vicissim (adv)	vicissem. (vicinor,v,0)		sp
4	479	.	.	.	sensus	sensu		≈
4	486	.	.	.	poterunt	poterit		≈
4	491	.	.	.	seorsum ? (1,adj)	videri. (video, 347)	???	≠
4	493	.	.	.	?…?	necessest.		?…?
4	495	.	.	.	nascuntur	Nascantur		≈
4	496	.	.	.	possint	possunt		≈
4	498	.	10	2	aequa (aequo,v,2)	Aeque (adv,10)		ok
4	500	.	.	.	poterit	poteris		≈
4	501	.	.	.	iuxtim quadrata	iustim quadratum		sp?
4	502	.	.	.	egentum (egeo)	egentim.		sp
4	514	.	316	215	si	sibi		ok
4	517	.	70	4	prava	Parva	prava	ok
4	526	.	.	.	corpoream	Corporea		≈
4	532	.	.	.	expleti (expleo,10)	explexis		sp
4	543	.	.	.	murmure (n)	murmura		≈
4	544	.	.	.	reboat (2)	revorat		sp
4	544	29	.	26	regio	retro (adv)	retro	ok
4	545	.	.	.	[et cycni tortis] convallibus (n) [ex]	[Et] validus (adj) [necti tortis ex]		=?
4	548	.	63	6	emittimus	mittimus	emittus	ok
4	549	.	22	32	verborum	nervorum	verborum	ok
4	552	.	.	.	levor levore	levo letiore		sp?
4	553	.	173	98	una	illa	una	ok
4	567	.	.	.	verbi	verbis		≈
4	570	.	.	.	locis	lopis		sp
4	576	.	.	.	magna	magnus		≈
4	577	.	37	60	[vidi] reddere [vocis]	[videre] odore [vocis].		ok
4	579	50	.	76	docta (doceo)	dicta (dico)	dicta	ok
4	581	.	.	.	faunos	faunes		sp
4	582	.	26	1	iocante (iocor,1)	locanti. (loco,26)	iocanti	ok
4	587	.	.	.	velamina	ullamina		sp
4	590	33	28	113	cetera	Petere (peto)	genera	ok
4	594	1	.	1	miraculorum	auricularum.	auriculorum	ok
4	598	103	.	374	[saepe ibi] demus (do)	[saepe] videmus.	videmus	ok

4	600	.	.	.	renutant	renuntant.		sp
4	603	.	.	.	cunctas	cuncta		≈
4	604	.	.	.	ubi una	ubina.		=?
4	608	10	.	1330	feriunt (ferio,10)	fuerunt (sum,1330)	fuerunt	ok
4	609	.	.	.	viis	vis		sp
4	611	.	18	4	[saepem] intra (1+3)	[Saepe] supra	intra	ok
4	611	0	122	6	saepem [intra] (n/adj)	Saepe [supra]	saepta	ok
4	615	.	411	395	Nec	Hoc		ok
4	616	.	38	1	operaeve (-ve) (≠opus)	opere. (opus)		ok
4	622	.	.	.	manantis	manantes		≈
4	624	.	.	.	umida lingua	Umidai lingua		≈
4	624	2	.	5	sidentia (sido)	sudantia (sudo)	sudantia	ok
4	631	.	.	.	possis	posses.		≈
4	632	0	5	1	umidulum	umidum (humidus)	umectum (humectum)	ok
4	633	.	.	.	unicus aptus ?	ut videamus.		=?
4	636	.	.	.	?…?	est.		≠
4	637	.	217	19	ali [cibus] (alo,v)	aliis cibus	ali	ok
4	637	.	10	1330	fuat (sum,1330)	fruat (fruor,10)	fuat	ok
4	638	45	10	1	aliquae	itaque	utique	ok
4	641	.	.	.	coturnicibus	cocturnicibus (1)		=
4	642	.	508	314	id	ut		ok
4	642	.	314	508	ut	Id		ok
4	648	.	.	.	variante	variantque (vario)		≠
4	648	349	.	1238	ex	et	et	ok
4	659	.	.	.	corpora	corpore.		≈
4	660	.	2	8	caulas	caudas	caulas	ok
4	662	.	.	.	mirum	nimorum		sp
4	668	.	.	.	? ut ?	Fit		=?
4	677	.	.	.	alius…et ?	alius et		~
4	680	.	.	.	volturniique	Volturnique		sp
4	681	1	.	4	permissa	promissa	promissa	ok
4	682	76	.	25	dicit	Ducit	ducit	ok
4	696	.	.	.	redolere	redolore		sp
4	698	.	.	.	creatum	creatam.		≈
4	710	.	.	.	quin	Qum		sp
4	710	.	.	.	explaudentibus	explondentibus		sp
4	712	.	8	2	rabidi	rapidi	rabidi	ok
4	719	.	.	.	illis	ilus.		sp
4	721	.	2	77	ulla	villa	ulla	ok
4	730	.	10	25	rara (adj,25)	ara (n,10)		ok
4	735	.	.	.	omne genus	Omnigenus		=?
4	736	.	.	.	fiunt	flunt (≠fluo)	fiunt	sp
4	740	.	117	6	animalis	anima.	animalis	ok
4	741	.	.	.	casu atque hominis	atque homines casu		vv
4	743	.	.	.	tenvia	tenua		sp
4	752	.	.	.	quoniam docui	docui quoniam		vv
4	752	.	.	.	leonem	leonum		≈
4	755	.	.	.	leonem et cetera	leonum cetera		≈
4	756	.	.	.	atque oculi	oculi atque		vv
4	760	.	1191	970	quem	que. (med. quae)		ok
4	766	.	1	33	letique	litique. (litis)		ok
4	791	14	.	4	referunt	repetent	repetunt	ok
4	795	.	5	52	sentimus	Consentimus	sentimus	ok
4	798	.	.	.	locis	locos		≈
4	798	.	6	1330	sint	sin		ok
4	802	312	.	1200	nisi] se (312)	nisi] que (med.=quae,1200)		ok
4	804	.	349	162	ad	ex	ad	ok
4	811	.	3	23	noscere	nocere	noscere	ok
4	815	10	95	3	praeter (8+2)	Praeterea	praeterquam	ok
4	818	.	161	281	non	nos		ok
4	820	.	.	.	vir tuti	virtuti		=?
4	822	0	.	5	[his vitium] vementer [rebu’ necesse	[his rebus vitium] vehementer [ine	vehementer	ok
4	822	1330	.	14	[his vitium vementer rebu’] necesses	[his rebus vitium vehementer] ines	inesse	ok
4	823	.	.	.	vitareque	ultareque		sp
4	825	.	.	.	possemus	possimus		≈
4	825	.	.	.	?…?	via.		≠
4	829	.	0	6	bracchia	Bacchia (0)		ok
4	836	.	.	.	natum	nata.		≈
4	843	.	.	.	conferre (9)	consere		sp
4	847	.	.	.	parmai (n)	parmat		sp
4	856	.	.	.	procul	pocul		sp
4	876	.	.	.	ieiuna	ieluna		sp
4	877	.	.	.	queamus	quemus		sp
4	877	.	2	228	fiat (fio)	flat (flo)		ok
4	878	.	.	.	varieque (adv)	vareque		sp
4	879	.	.	.	oneris (onus)	onoris		sp?
4	885	.	.	.	constat	constare		≈
4	886	.	26	41	ergo	Ego		ok
4	889	.	.	.	factu	factum		≈
4	890	.	34	10	ferit (ferio)	perit	ferit	ok
4	897	.	.	.	?…?	utac		=?
4	899	.	.	.	tantula	Tantuia		sp
4	905	.	.	.	pondera magna	pondere magno.		/≈
4	907	.	.	.	modis	modi		≈
4	907	.	.	.	quietem	quiete.		≈
4	909	.	.	.	suavidicus	Suautdicis		sp
4	915	.	.	.	tutemet	Tutimet		≈
4	928	.	.	.	posset	Possit		≈
4	929	.	4	5	confiat	conflat	confiat	ok
4	934	41	.	314	atque [ab] ibis	atque eius	atque eius	ok
4	936	.	2	1	callo	gallo	callo	ok
4	945	.	.	.	eiciatur	Eliciator		sp
4	959	.	208	27	partim (adv,27)	parte (n,208)		ok
4	961	2	.	26	actus (≠ago)	intus. (adv,23+3)	intus	ok
4	982	24	5	2	concessum (24)	consensum (consentio,5)	consessum	ok
4	983	.	.	.	scenaique (scena,3)	Scenatque		sp
4	984	.	10	25	voluptas (25)	voluntas.	voluptas	ok
4	989	.	.	.	palma	palmas		≈
4	990	.	.	.	?…?	saepe quite.		=?
4	991	.	.	.	vocesque	vocisque (vox)		≈
4	995	.	.	.	fugae	fuga		≈
4	995	.	.	.	dedita	ddita		sp
4	996	.	12	6	erroribus	terroribus	erroribus	ok
4	1011	80	49	104	[qui] mentibus	[que] montibus	motibus NB	ok
4	1013	.	.	.	expugnat	exipugnat		sp
4	1022	16	.	1	externantur	Exterruntur	exterrentur	ok
4	1026	.	.	.	lacum (8)	iacum		sp
4	1026	.	.	.	sei (poetic)	se		≈
4	1032	62	.	161	quodam	quoque.	quoque	ok
4	1033	.	.	.	coloris	colores.		≈
4	1034	.	1191	1306	qui	Quae		ok
4	1035	349	1238	508	ex	Et	ut NB	ok
4	1036	.	.	.	cruentent	cruentet.		≈
4	1038	.	.	.	adulta	advita		sp
4	1058	5	.	26	momen	nomen	nomen	ok
4	1059	.	173	2	illaec (illic,adj)	ille		ok
4	1061	7	.	3	aves (7)	ames	ames	ok
4	1061	.	1330	215	nam si	Namst (-est)		ok
4	1065	.	11	11	conlectum (10+1)	coniectum	conlectum	ok
4	1068	.	0	6	ulcas (ulcus,6)	Vicus (vicus,0)	ulcus	NB
4	1083	.	173	2	illaec (illic,adj)	illae		ok
4	1085	.	0	8	refrenant (8)	frenant (0)	refrenat	NB
4	1089	1233	.	1191	quo [mage plurima] (adv)	cuius [quam plurima]	cuius	ok
4	1089	4	.	1361	[quo] mage [plurima] (adv)	[cuius] quam [plurima]	quam	ok
4	1096	.	93	80	mentem ?	vento	vento	ok
4	1098	.	.	.	membris	membri		≈
4	1115	.	11	11	conlecta (10+1)	coniecta	conlecta	ok
4	1118	.	1233	253	quid	quod		ok
4	1121	.	.	.	viris (20)	urtris		sp
4	1123	.	0	2	babylonica	babylonia (0)		NB
4	1124	.	.	.	vacillans	vigillans.	vacillans	sp
4	1125	9	.	4	argentum	Unguenta	unguenta	ok
4	1129	.	.	.	fiunt (228)	flunt		sp
4	1130	.	.	.	alideusia Ciaque (poetic?/)	alidensia chiaque		sp/sp?
4	1131	.	.	.	lychni (1)	luidi.		sp
4	1137	.	.	.	ambiguo (1)	ambiguo (ambi+quo?)		??
4	1141	.	6	34	mala	male (adv)		ok
4	1145	.	.	.	inliciaris (poetic? illicio,1)	inligniaris.		sp
4	1152	.	.	.	petis	ppetis		sp
4	1152	.	508	153	Aut	Ut	aut	ok
4	1154	.	314	382	his	is		ok

4	1156	.			0		3	deliciis (delicia)	delictis (delinquo pt)		NB
4	1160	.		.		.		fetida	foetida		=
4	1168	.		.		.		Lamia	iamina		sp
4	1170	.		.		.		est [si]	esti; (w/ colon;poetic? ≠edo?)		sp
4	1174	.		.		.		turpi	turpis		≈
4	1176	.			20		62	longe fugitant (adv/v)	longi fugitan (adj/sp)		ok/sp
4	1180	.			63		15	ammissu (amito,15)	missum (mitto)	ammissum	ok
4	1182	.		.		.		cadat (cado)	cadet		≈
4	1183	.		.		.		stultitiaeque	Stultitiaque		≈
4	1186	.		.		.		poscaenia	postcaenia		=
4	1188	.		.		.		possis	posses		≈
4	1198	.		.		.		possunt	possent		≈
4	1200	.		.		.		salientum (2)	sallentum		sp
4	1200	.		.		.		retractat	retractant.		≈
4	1202	.		.		.		vinclis (vinculum,8)	vinciis		sp
4	1202	.			25		29	vinxit (vincio)	Vixit (vivo)		ok
4	1203	.		.		.		?quam?	cum		=?
4	1209	.		.		.		vicit	ulcit.		sp
4	1212	.			208		4	patribus	partibus	patribus	ok
4	1220	.		.		.		multa modis	multimodis		~
4	1222	.		.		.		ab	a		=
4	1224	.		.		.		refert (refero+)	refer		sp
4	1230	.		.		.		quod cumque	quo cumque	quodcumque	≈
4	1234	.			10		12	pater	praeter		ok
4	1243	.			43		18	cedit	credit	cedit	ok
4	1244	.			411		382	his	hic		≈
4	1247	.			6		8	muliebri (adj)	mulieri (n)		ok
4	1252	.		.		.		post	pos		sp
4	1255	.		.		.		uxores	Uxoris		≈
4	1259	.		.		.		conveniant	conveniunt		≈
4	1262	.		.		.		aliis	alii		≈
4	1262	.		.		.		tabentque	tabenque		sp
4	1267	.		.		.		lumbis (lumbi)	lumbris. (lumbare?)		sp
4	1268	.			8		395	nec	Ne (≠-ne)	nec	ok
4	1275	.		.		.		gravidaeque	gravidaque.		≈
4	1281	.			13		50	modis	moris	modis	ok
5	2	.			381		382	maiestate hisque (his)	maiestatis atque		≈/ok
5	5	.		.		.		quesitaque	quaesita		≈
5	12	.			18		26	locavit	vocavit (voco,vocavi)		ok
5	14	.			39		4	Ceres (4)	geres (39)		ok
5	30		7	.			161	aves Stymphala (1)	nobis tymphala	nobis	ok/sp
5	31	.		.		.		Thracam (1)	Thracia		≈
5	33	.		.		.		acerba	acerban		sp
5	34	.		.		.		stirpem	stirpes		≈
5	35	.		.		.		Atlanteum (1)	atianeum		sp
5	35		0	.			4	[pelageque] sonora	[pelegique] severa.	severa	sp/ok
5	38	.			4		29	victa (vinco)	vincta (vincio)		ok
5	38		312		133		215	sei (se)	Sed	si NB	ok
5	44	.		.		.		tumst (est)	sunt		/≈
5	45	.		.		.		cuppedinis	cuppedines		≈
5	53	.			349		150	de	e		ok
5	61	.		.		.		incolumis	incolumne (+ne)		sp?
5	67	.		.		.		materiai	materiam.		≈
5	70	.			6		1330	sint	sin		ok
5	71	.		.		.		loquella	loquela.		=
5	85	.			33		14	aetheriis (aetherius)	aetheris (aether)		ok
5	100	.		.		.		insolitam	insolitum		≈
5	114	.		.		.		religione	Relligione		sp
5	116		15		15		41	manare	meare. (meo)	manere NB	ok
5	117	.			208		25	par	pars		ok
5	121	.		.		.		sermone	sermoni		sp
5	122	.		.		.		numine distent	niminbistet.		r
5	131	.		.		.		crescat et insit	crescit et inestit.		≈/=/sp
5	133	.		.		.		neque a nervis	neque arvis (aruum)		r
5	133		1	.			82	longiter (adv)	longius (comp adj)	longius	ok
5	134	.		.		.		quid	Quod		≈
5	142	.		.		.		glebis (glae-)	gleberis		sp
5	152	.			215		25	quod tangi	quod si		ok
5	154		215	.			150	[tenuest] si [corporu' deorum]	[tenues] de [corpores eorum.]	de	ok
5	154		34	.			314	[tenuest si corporu'] deorum	[tenues de corpores] eorum.	eorum	ok
5	175		114	.			26	At	An	An	ok
5	182		34	.			33	dis	divis (30+3)	divis	ok
5	185	.		.		.		sese	se		=
5	187	.		.		.		multa modis	multimodis		r
5	191	.		.		.		possent	possint.		≈
5	193	.			6		7	meatus (7)	maestus. (6)		ok
5	201		45	.			8	aliquam (aliquis,45)	avidam (avidus,8)	avidam	ok
5	208	.		.		.		bidenti	dibenti.		sp
5	209	.			9		4	aratris	atris. (ater,9)		ok
5	211	.		.		.		terraique	Terraeque		≈
5	223	.			4		3	indigus	indignus		ok
5	227	.		.		.		restet transire	reet transirest		sp/~
5	230	.		.		.		almae	Arme		sp
5	230	.		.		.		loquella	loquela.		=
5	239	.		.		.		eodem	eadem		≈
5	241	.		.		.		nativo	navitom		sp
5	245	.			104		64	item	idem		ok
5	251		93		77		2	nulla	ulla	nonnula NB	ok
5	264	.		.		.		quicquid	quidquid		≈
5	272	.			15		52	via [secta semel]	semel [secta semel]		ok
5	274	.			33		4	horas	oras.		ok
5	282	.			5		32	recenti (recedo+recens)	regenti (rego)		ok
5	288	.		.		.		disperit	disperis		≈
5	291	.			508		1238	et	Ut		ok
5	293	.		.		.		suppeditet	suppedite		≈
5	295	.		.		.		lychni	lyclini		sp
5	297	.			4		2	properant	proferant (profero,4)		ok
5	301	.			2		9	celatur	celeratur		ok
5	302	.		.		.		putandumst	putandum.		≈
5	304	.		.		.		quicquid	quidquid		≈
5	310	.			0		12	foedera (12+0)	foederant (foedero,0)		ok
5	312	.		.		.		quae fore	Querare		=?
5	312	.		.		.		vetitumque	sibi cumque		=?
5	318	.		.		.		omnem	omne.		≈
5	326	.		.		.		funera	funara (≈funarius)		sp
5	327	.			19		217	alias	ali		ok
5	331	.		.		.		naturast mundi	Natura mundist		vv
5	339	.		.		.		periisse (pario,perior,poetic?)	perisse		sp?
5	342	.			114		381	atque	at		ok
5	359	.			1330		228	fit	sit		ok
5	367	.			3		32	coorta (coorior)	coperta. (cooperio)		ok
5	375	.			349		1238	et	e		ok
5	377	.		.		.		mortali	mortalis		≈
5	382	.			1		173	ollis (ollus=ille)	olis. (olo=oleo)		ok
5	396	.			0		2	superat et lambens	superavit et ambens (ambio,0)		ok
5	397	.		.		.		Phaethonta	petontana		sp
5	399	.			413		104	tum	cum		ok
5	400	.			30		42	fulminis	fluminis		ok
5	405	.			2		10	Graium	gratum		ok
5	410		1238	.			153	et	Aut	aut	ok
5	412	.			35		16	urbis	undis.		ok
5	425	.		.		.		coire	cotre		sp
5	428	.		.		.		omne genus	Omnigenus		r
5	429	.		.		.		conveniant	conventa		≈
5	430		52	.			122	semper	saepe.	saepe	ok
5	430	.			33		228	fiunt	fluunt		ok
5	431	.		.		.		terrai	Terrae		≈
5	433	.		.		.		altivolans	Alte volens		=?
5	437	.			0		80	inde (80)	indue (induo,0)		ok
5	439	.		.		.		magnas	magna		≈
5	440		150	.			349	[omne genus] de	[Omnigenus] e	e	ok
5	441	.		.		.		vias	via		≈
5	447	.		.		.		umore	umor		≈
5	449	.		.		.		terrai	terrae		≈
5	458	.		.		.		=?	et		=?
5	460	.		.		.		videntur	videmus		≈
5	463	.		.		.		exalare	Exalantque		≈
5	468	.		.		.		undique flexit	saepsit		=?
5	472	.		.		.		interutraque	Inter utrasque		r
5	476	.		.		.		interutraque	inter utrasque		r
5	481	.		.		.		plaga	plage		sp

5	482	.			0		5	salso suffudit (suffundo,5)	salsos offudit (offundo,0)		NB
5	485		177	.			162	a [limini' parte]	ad [limina partem.]	ad	ok
5	491		5	.			1	densebant (denseo,5)	Densabant (denso,1)		ok
5	500	.		.		.		leviora (levis comp)	levior		≈
5	503	.		.		.		commiscet	Commisci		≈
5	503	.		.		.		hic	haec		≈
5	507	.		.		.		Pontos	ponto		≈
5	513	.		.		.		??=	eodem.		=?
5	515		43	.			153	hinc	Aut	aut	ok
5	515	.			1275		1306	qui	quis		ok
5	516	.		.		.		fluvios	fluvius		~
5	518	.			0		10	lucida (lucidus,10)	lucia (=fish,if≠proper noun,0))		ok
5	521	.		.		.		immania	summania		sp
5	524		10	.			41	aventis (aveo)	euntis. (eo)	euntis	ok
5	528	.		.		.		creatis	creati.		≈
5	530	.		.		.		omne	omnem.		≈
5	531	.		.		.		siet	sit et		r
5	532	.			14		1	vegeat	vigeat		ok
5	533	.		.		.		pedetemtim progredientis	pedetemti progredientes		sp/≈
5	536	.			24		19	supter (=subter)	super		ok
5	537	.		.		.		ineunte (ineo)	ineunt		≈
5	538		50	.			25	crevit (cresco,crevi=creti)	vivit.	vivit	ok
5	545	.		.		.		quaeque	quaeat		sp
5	545	.			152		198	magni	magi		ok
5	551	.		.		.		supra	supru		sp
5	553	.			108		8	aeriis (aerius,8)	aeri (aer,108)		ok
5	555	.			6		19	apta	aucta.		ok
5	558	.			5		19	apta	rapta (rapio,part.)		ok
5	559	.		.		.		pernici	pernice		≈
5	560		150	.			117	animi	animae	animae	ok
5	560	.			1275		253	quid	Quis		ok
5	563	.		.		.		coniunctis	Coniuncta		≈
5	567	.			0		1	adicere	Adlicere (allicio,3rd,0)		NB
5	568	.		.		.		nil ea is his intervallis	Nihil nisi intervallis		=?
5	568	.			0		5	libant (5)	librant. (libro,0)		NB
5	570		5	.			4	mulcent	fulgent.	fulgent	ok
5	571	.		.		.		filumque	ilumque		sp
5	576	.		.		.		suam	sua		≈
5	580	.			108		8	aera (aera)	Aere (aer)		ok
5	581		8	.			26	minui (minuo)	mi (ego)	mi<nui>	ok
5	584	.		.		.		quantaque quantast	Quanta quoque quantast		=?
5	589	.		.		.		absunt	absit.		≈
5	595	.		.		.		parte	perte		sp
5	599	.		.		.		vaporis	vapore.		≈
5	605	.		.		.		percipiat	percipitat		sp
5	606	.			9		67	ita est	sita est (situs,adj)		ok
5	610		349	.			1238	e	et	et	ok
5	613	.		.		.		aestifer ut	Aestiferiu		sp
5	614		0	.			17	rellata	recta	recta	ok
5	617	.		.		.		cancri se ut	Canceris ut		r
5	632	.			138		27	etenim	etiam		ok
5	648	.		.		.		illa	ille		≈
5	651	.		.		.		ultima	vetima		sp
5	654	.		.		.		convortere	convortore		sp
5	656	.			6		1	roseam Matuta	rosea matura (subst.adj)		≈/ok
5	657	.			349		1238	et	e		ok
5	667	.		.		.		possunt	possit.		≈
5	675	.			30		42	fulmine [postremo]	Flumina [postromo]	fulmina	ok/sp
5	679	.		.		.		consequë (NB: ë)	Consequiae (n,0)	consecue	≈? NB
5	679	.			628		24	redeunt (24)	rerum (628)		ok
5	684	.			349		1238	et in	e in		ok/=
5	690	.		.		.		metans (meto)	metas		≈
5	692	.			1		5	concludit (5)	contudit (?contundo,1)		ok
5	693	.		.		.		obliqui (obliquus)	Obliquo		≈
5	700	.			76		39	diei (39)	dici (76)		ok
5	705	.			3		7	percussa (percutio)	perculsa (percello)		ok
5	708	.		.		.		?pleno bene?	beneno		=?
5	711	.			51		138	iam	tam		ok
5	713	.		.		.		pilai	pilae.		≈
5	720		1330	.			215	sit	si	si	ok
5	727	.		.		.		Babylonica	babylonisa		sp
5	730	.			0		173	illo	ilio (ile,...il_,0)		NB
5	737	.		.		.		Venus	veneris		≈
5	737	.			2		87	ante	algi.		ok
5	738	.		.		.		zephyrus	zephyri		≈
5	742	.		.		.		pulverulenta	Pulverunta		sp
5	747	.			43		2	crepitans	creditans (credo)		ok
5	747		8		25		60	prodit (prodo)	Redit (redeo,25)	reddit NB	ok
5	750	.		.		.		fieri	fieri		sp
5	753	.			517		125	solis	possis. (possum)		ok
5	756	.		.		.		eodem	eadem		≈
5	761	.		.		.		perire	periri		≈
5	764	.			0		4	perlabitur	periabitur (perior,0)		NB
5	768	.		.		.		fulgit	fulget		≈
5	782	.		.		.		tollere	Tolleret		≈
5	782	.			43		97	crerint (creo)	credunt (credo)		ok
5	784	.		.		.		camposque	campusque		≈
5	790	.		.		.		virgultaque	vigultaque		sp
5	799	.			1		104	tum	tus (=thus)		ok
5	800	.		.		.		maiora	maiore		≈
5	802	.		.		.		relinquebant (relinquo)	reliquebant		sp
5	805		16	.			63	passim	primum (adv)	primum	ok
5	809	.			0		37	aestus (-us)	aestas. (-as,0)		NB
5	812	.			508		1238	et	Ut		ok
5	823	.			117		21	animal	anima		ok
5	824	.		.		.		magnis	magni		≈
5	825	.		.		.		aeriasque	Aeriaeque		≈
5	833		2		50		1	clarescit	crescit	succrescit NB	ok
5	836	.		.		.		possit	potuit		≈
5	838	.			124		15	facie (n)	facit (v)		ok
5	839	.		.		.		androgynum interutrasque	Androgynem inter utras ...		r
5	839	.			25		9	[nec utrum] utrimque	[... ned utramque] utrumque		ok
5	841	.			274		9	muta	multa		ok
5	844	.			31		1330	foret (sum)	volet (volo2)	foret	ok
5	846	.		.		.		absterruit	abserruit		sp
5	850	.			0		5	procudere	procludere (0)		NB
5	851	.		.		.		primum [ut]	primunt (primo=adv)		sp
5	852	.		.		.		possint	possis		≈
5	852	.		.		.		remissa	remissis.		≈
5	853	.		.		.		maribus	marius (if≠name)		sp
5	853		10	.			60	[possit] avere	[possis] habere	habere	≈/ok
5	854		30		14		42	[mutuaque] insinuent	[Mutua qui] metuent	mutent (muto)	ok
5	859	.			1		3	tutata (tutor)	tuta (tuto,part.)		ok
5	865	.			6		2	veterino (veterinus)	veteri non (vetus)		ok
5	866	.		.		.		bucera	bugera		sp
5	868	.		.		.		secuta	secutae		≈
5	871	.			4		95	nil (=nihil,95)	ni (4)	nil	sp
5	873	.		.		.		quare	quari		sp
5	881	.			208		70	partis (208) [ut si par]	parvis (70) [ut non pars][?]		ok
5	881		25	.			208	[partis ut si] par	[parvis ut non] pars	pars	ok
5	884		5		5		3	quaquam (=quoquam in Lu.. 5)	quamquam (5)	haudquamquam	ok
5	885	.			1		1	lactantia (lacto, 1)	laetantia (=laetans,pt)		ok
5	888	.		.		.		florente	florenta		sp
5	888	.			0		26	pueris	puerili (adj,0)		NB
5	889	.			10		1	Occipit	Officit		ok
5	892	.			8		2	rabidus	rapidis		ok
5	896	.			1		6	proiciunt	proficiunt		ok
5	904	.		.		.		una	unam.		≈
5	906	.		.		.		flaret	flare		≈
5	906	.			109		33	foras (33, adv)	feras (fero, 109)		ok
5	913	.		.		.		hominem	homine		≈
5	914	.			45		18	ponere (pono, v. 18)	pondere (pondus, n. 45)		ok
5	923		198		215		628	sed vis	Sed si	sed res NB	ok
5	925	.			1227		114	At	Et		ok
5	933	.			0		4	aratri (aratrum, 4)	arari. (aro,0)		NB
5	934	.		.		.		molirier	mollerier		sp
5	935	.		.		.		virgulta	virguita		sp
5	937	.		.		.		crearat	crearant.		≈
5	944	.			3		15	dura (durus, 15)	dira (dirus, 3)		ok
5	947	.		.		.		clarigitat late	Claricitatiate		r
5	948	.		.		.		vagi	vagis		≈

5	949	.	.	.		umori'	umore		≈
5	953	.		0	32	scibant (scio, 32)	scribant (scribo, 0)		NB
5	959	.	.	.		scibant	sciebant		=
5	962	.		2	17	iungebat	Iugebat		ok
5	970	.	.	.		nuda dabant	Nudabant		r
5	973	.	.	.		pavidi (pavidus, 5)	parvidi		sp
5	976	.	.	.		rosea	rotea		sp
5	977	.	.	.		a parvis	Apervis		r,sp
5	984	.		0	15	eiectique (eicio, 15)	Electique (eligo,0)		ok
5	985	.	.	.		validive (-ve)	validique (validus, 45,+que)		≈
5	989		3 .		1	labentis (labo part.)	lamentis (lamenta, 1)	lamentis	ok
5	992	.	.	.		gemitu	gemitus		≈
5	993	.		6	2	vivo (vivus)	vino (vinum)		ok
5	995	.	.	.		??tetra??	vicerat		=?
5	996	.	.	.		accibant	accibunt		≈
5	997	.		96	22	donique (donec) (conj)	Denique (adv)		ok
5	1001		0 .		1	fligebant (fligo)	lidebant (lido)	lidebant	ok
5	1002	.		395	382	hic	Nec		ok
5	1003	.	.	.		saevibat	Saevidat		sp
5	1003	.		2	18	ponebat (18)	potebas (2)		ok
5	1006	.	.	.		[missing line]	Improba … iacebat.		=?
5	1008	.	.	.		dabat	daeant (word?)		sp
5	1009	.		1	1	inprudentes	prudentes	iMprudentes	ok
5	1011	.	.	.		pellis	pellus		sp
5	1011	.		8	2	casas	cassas		ok
5	1013	.	.	.		conubium	Cognita sunt		=?
5	1016	.	.	.		ferre	ferri.		≈
5	1020	.	.	.		violari	violare.		≈
5	1023	.	.	.		omnis	omni.		≈
5	1025	.	.	.		caste	casti.		≈
5	1033	.	.	.		vim	vis		≈
5	1033	.	.	.		quo ad	quod	quoad NB	r
5	1035	.	.	.		infestus	infessus		sp
5	1038	.	.	.		etiam	tiam		sp
5	1039	.	.	.		porro	proporro		sp
5	1048	.	.	.		utilitatis	Utilitas		≈
5	1049	.	.	.		scirent	sciret		≈
5	1049	.	.	.		viderent	videret.		≈
5	1052	.	.	.		surdis	surdes.		≈
5	1053	.		215	1330	facilest (+est)	[facile] si		ok
5	1055	.	.	.		sonitus obtundere (obtundo,5)	sonitu subtundere (sub+tundere)		sp
5	1058	.	.	.		varia res	varias res		≈
5	1062	.		900	314	id	in		ok
5	1063		9 .		198	inmane (=immane,9)	magna (198)		ok
5	1064	.		17	2	fremunt	premunt		ok
5	1065	.	.	.		alio	alia		≈
5	1065		0 .		1	restricta [minantur](restringo,0)	stricta [minantur].(stringo,1)	stricta	ok
5	1067		1227 .		114	et	At	at	ok
5	1068	.		0	23	iactant	lactant (=lacio,0)		ok
5	1068	.		517	23	petentes (peto)	potentes. (potens,5,possum,)		ok
5	1069		8 .		8	minitantur	imitantur	imitantur	ok
5	1071	.	.	.		deserti baubantur	desertibus aubantur		sp
5	1072	.	.	.		plorantis	Florantis		sp
5	1076	.	.	.		?ubi? =?	sub		=?
5	1082	.		0	4	praedaeque (4)	praedataque (praedo,0)		ok
5	1084	.		1238	508	ut	et		ok
5	1085	.	.	.		greges	gregis		≈
5	1087	.	.	.		varii	variis		≈
5	1088	.		274	9	muta	Multa		ok
5	1090	.	.	.		res	re		≈
5	1094		0 .		2	inlita	insita	insita	ok
5	1095	.	.	.		vapore	vaporis.		≈
5	1096	.		508	1238	et	Ut		ok
5	1097	.	.	.		aestuat	Aestua		≈
5	1097	.		34	20	arboris	ardoris		ok
5	1099	.		2	3	et micat	Emicat		ok
5	1101	.	.	.		ignem	igne.		≈
5	1102	.		0	44	quoquere … vapore	quoq. vere (veru,0)		NB
5	1105	.	.	.		Inque	in		≈?
5	1106		0 .		160	benigni (adj)	[et] igni.	igni	NB
5	1110	.	.	.		pecus	pecedues	pecuA	sp
5	1110	.		77	3	[divisere atque] dedere	[diviser atque] debere.		ok
5	1112		0 .		14	vigorque	vigebant. (vigeo)	vigebant	ok
5	1116	.		1	59	creti (cresco,50)	certi. (certus)		ok
5	1121	.		1	2	stabili (stabilis,2,adj)	stabuli (stabulum,1,n)		ok
5	1122	.	.	.		placidam possent	placida possunt		≈/≈
5	1124	.		120	10	[certantes] iter (n)	[Certantesque] inter		ok
5	1126	.	.	.		taetra	thetra.		sp
5	1129	.		0	55	sine	side (0)		NB
5	1130	.	.	.		ambitionis	ambitiones.		≈
5	1132	.		46	217	aliis	altis		ok
5	1141	.	.	.		itaque	taque		sp
5	1141	.		3	22	redibat	recidat.		ok
5	1142	.		11	28	petebat	patebat.		ok
5	1143	.		9	97	creare	recreare.		ok
5	1145		65	0	7	vi colore (65)	vi cere (0)	colere (7)	NB
5	1147	.		0	4	iura	Iura. (Lura,0)		NB
5	1150	.		65	7	colere (colo)	colore		ok
5	1152	.	.	.		iniuria	iniuriam		≈
5	1152	.		4	198	vis	ius		ok
5	1159	.	.	.		delirantes	dilirantes		sp
5	1172	.	.	.		propterea	proptere		sp
5	1178	.		173	77	ulla	illa		ok
5	1184	.	.	.		varia	varias		≈
5	1185	.	.	.		fieret	fierent		≈
5	1189		39 .		28	lux	nox	nox	ok
5	1190		9 .		4	serena	severa.	severa	ok
5	1190	.		349	1248	et nox	e noctis		ok/≈
5	1192	.		30	42	fulmina	flumina		ok
5	1198	.	.	.		ullast velatum	ulla velatumst		≈
5	1203	.		3	3	pacata (pacatus,paco part.)	placata (placo,part.)		ok
5	1208	.	.	.		erigere	eriger		sp
5	1214	.	.	.		solliciti (8)	Et taciti. (tacitus,)		≠
5	1220	.	.	.		fulminis	Fulmini		≈
5	1221	.	.	.		murmura (+murmuro?)	murmure (murmur)		≈
5	1224	.	.	.		nequid	Nequod		≈
5	1225		3	1	1	adultum	adauctum. (adauctus)	adactum	ok
5	1226	.	.	.		summa	Summe		sp
5	1229	.		177	120	ac	a		ok
5	1232	.	.	.		ad vada	aquada		r
5	1236	.		316	35	sub	sui		ok
5	1241	.		10	11	[est aes] atque	[est] aeque (adv)		ok
5	1243	.		20	20	ingentis	gentis		ok
5	1248	.	.	.		pinguis	panguis		sp
5	1252	.	.	.		quidquid	Quicquid		≈
5	1253	.	.	.		sonitu	sonitus		≈
5	1254	.	.	.		ab	A		=
5	1255	.	.	.		venis	venit		≈
5	1258	.	.	.		terra	terras		≈
5	1259	.		35	33	capti (capio part.,33)	capiti (caput,35)		ok
5	1260	.	.	.		videbant	videbat		≈
5	1266	.	.	.		possent	possint.		≈
5	1266	.		2	103	darent (do)	parent (pareo,2)		ok
5	1266	.		1238	508	ut	et		ok
5	1267		28 .		1	domo levare	dolaret levare	dolare et	ok
5	1272	.	.	.		poterat	poterant		≈
5	1273	.		180	104	tum fuit	Nam fui		ok/≈
5	1280	.	.	.		laudibus	Claudibus		sp
5	1285	.	.	.		flamma	flamm		sp
5	1288	.	.	.		maior	maiore.		≈
5	1294	.	.	.		species est	speciesest		r
5	1294	.		1	3	ahenae (aenus)	athenae. (1)	ahenae	ok
5	1300	.	.	.		biiugos	biiugo		≈
5	1302	.	.	.		lucas	cas		sp
5	1302	.	.	.		tetras	tetros.		sp
5	1307	.	.	.		belli	bellis		≈
5	1315	.	.	.		≠	Terrificas … cristas.		=?
5	1319	.		11	28	petebant	patebant.		ok
5	1325	.		80	3	minitante (minitor,3)	mente		ok
5	1328	.	.	.		≠	In se …tela.		=?
5	1330	.		2	2	adactus (adigo,2)	adauctus (adaugeo,2)		ok

5	1331	.	.	.		petebant	petebat.		≈
5	1340		4 .		14	fata (fatum,4)	facta	facta	ok
5	1341		87 .		215	sic	Si	si	ok
5	1344	.	.	.		≠	Et magis … omni.		=?
5	1346	.	.	.		≠	Quam certo … orbi.		=?
5	1354	.	.	.		lanam	ianam (≠Jana)		sp
5	1361	.		177	114	at	A		ok
5	1366	.	.	.		defodere (defodio!)	defodire		sp
5	1368	.	.	.		terram	terra.		≈
5	1390	.	.	.		iuvabant	iuvabat		≈
5	1391	.	.	.		=?	omnia		=?
5	1392	.	.	.		gramine	gramina		≈
5	1393	.	.	.		propter	Proptere		sp
5	1397	.		61	1	ioca (1)	loca (locus,loca)		ok
5	1399	.		0	1	redimire (redimio,1)	redimere (redimo,0)		ok
5	1400	.		38	1	monebat	movebat.		ok
5	1404	.	.	.		vigebant	vigebat		≈
5	1405	.	.	.		somni	somno.		≈
5	1409	.	5 .		113	sonis	genus	genus	ok
5	1410	.	.	.		maiorem	Maiore		≈
5	1410	.	.	.		dulcedini' (poetic?)	dulcedine		=?
5	1416	.		0	173	[sic] illa	sigilla (0)		NB
5	1418	.	.	.		ferinae	ferina.		≈
5	1419	.		126	8	tunc	nunc		ok
5	1425	.		0	900	in	ina (0)		NB
5	1429	.	.	.		plebeia	plebela		sp
5	1436	.	.	.		versatili' (?poetic)	versatile		=?
5	1440	.	.	.		degebant	degebat		≈
5	1441	.	.	.		discretaque (discerno)	disceretaque		sp
5	1442	.	.	.		[puppibus et] res	[propter] odores.	†	≠
5	1442	.		104	138	iam	Tum		ok
5	1444	.	.	.		coepere	caepere		sp
5	1451	.	.	.		polire (polio)	polito. (polio)		≈
5	1456		33 .		374	conveniebat	corde videbant.	videbant	ok
6	1	.	.	.		aegris	aegros.		≈
6	4	.	.	.		solacia	solaci		≈
6	7	.	.	.		extincti	extincta		≈
6	10	.	.	.		mortalibus	acortalibus		sp
6	11	.	.	.		posset	possent		≈
6	13	.	.	.		excellere	excollere (≠excolere)		sp
6	14	.	.	.		corda	cordi		≈
6	16	.	.	.		passimque	Pausa atque		r
6	16	.	.	.		coegi	coget		≈
6	16		14 .		9	periclis	querellis	querellis	ok
6	17	.		1	9	vas	fas		ok
6	22	.	.	.		conspurcare	conspurgare		sp
6	28	.	.	.		recto	recta		≈
6	30	.		1330	228	fieret	fuerit		ok
6	31	.	.	.		[sic] natura	signatura (signo)		r
6	31		60 .		10	causa	casu	casu	ok
6	32	.		349	1238	et	E		ok
6	33	.	.	.		frustra	prustra		sp
6	34	.		16	25	volvere (25)	Volnere (volnus,16)		ok
6	35	.		150	33	caecis	caelis.		ok
6	45		23 .		228	fateare	fierique	fierique	ok
6	47	.	.	.		=?	insignem		=?
6	48	.	.	.		[ventosum et] certant	[Ventorum] exirtant …		sp
6	48		2 .		495	[plangentia] flamina	[… placentur] omnia	omnia	ok
6	49	.		0	4	furore	favore. (0)		NB
6	49		55 .		1330	sine	sint	sint	ok
6	51	.		49	80	mentibu'	montibus	mentibu'	ok
6	52		382 .		1238	haec	Et	et	ok
6	63	.	.	.		adsciscunt	adciscunt		sp
6	64	.	.	.		miseri	miseris		≈
6	68	.	.	.		longeque remittis	longique remitti.		sp/≈
6	71	.	.	.		violari	violaris		≈
6	71	.		2	16	oberunt	oderunt		ok
6	72	.		41	14	ira (n)	ire (eo)		ok
6	73	.	.	.		quietos	quietus.		≈?
6	74	.		0	20	fluctus	fletus. 0		NB
6	76	.		1330	108	feruntur	fuerunt.		ok
6	83	.		1238	1330	est [ratio fulgendi visque]	et [ratio caelisque tenenda.]		ok/=?
6	84	.		30	42	fulmina	flumina		ok
6	86	.	.	.		trepides	trepidas		≈
6	87	.	.	.		perveniret (-venio,-venire?)	perveneret (-veneo,-venire?)		sp?
6	91	.	.	.		fieri	fier		sp
6	92	.	.	.		calcis	callis.		sp
6	92	.		120	162	[praescribta] ad	[prescribita] ac		ok
6	95	.	.	.		laude	laudi		≈
6	102	.	.	.		praeterea	Pretere (med. version?)		sp?
6	102	.	.	.		nubes	nure.		sp
6	103	.	.	.		[lapides ac] tigna	[pepides ac] iigna		sp
6	105	.	.	.		ab bruto ?	avi		=?
6	110	.		0	8	malos (malus)	matos (=mattos, 0)		NB
6	111	.	.	.		petulantibus	petuiantibus		sp
6	112	.	.	.		chartarum	chatarum		sp
6	114	.	.	.		chartasve (-ve)	chartasque (-que)		≈
6	115	.	.	.		planguntque	plaguentque		sp
6	118	.	.	.		corpora	corpore		≈
6	120	.	.	.		exierunt (exeo)	exierum		~≈
6	122	.	.	.		tremere	teremere		sp?
6	124	.	.	.		conlecta	concollecta		sp
6	126	.	.	.		versanti	versante		≈
6	129		2	20	8	fissa (findo,part.)	missa (mitto part.)	scissa NB	ok
6	131		36 .		67	[saepe det] haut [parvum] (=haud)	[Saepe] ita [dat parvum]	ita	ok
6	132	.	.	.		perflant	perfiant.		sp
6	136	.	.	.		perflant	Perfiant		sp
6	141	.		25	2	evolvens (evolvo,2)	volens (25)		ok
6	144	.	.	.		aestus	aest.		sp
6	145		314 .		228	Id	Fit	fit	ok
6	149	.		28	1	propere	propter		ok
6	151	.	.	.		ingenti	ingentis		≈
6	151	.		10	37	repente (repente,adv,36,repens,1)	recente. (adj,=recens=adj,adv,9+1		ok
6	153	.	.	.		turbine (turbo)	Turne		sp?
6	154	.	.	.		?ulla?	vita		=?
6	155	.	.	.		sonitu	sonitum		≈
6	158	.	.	.		arto	artum.		≈
6	162	.	.	.		percutiat	Percuciat		sp
6	165	.		20	11	fulgere	Fugere		ok
6	168	.	.	.		ancipiti videas	Ungipiti videat		sp/≈
6	170	.		8	9	fulgorem (v)	fulgurem (n)		ok
6	178	.	.	.		ardescere	adescere (≠adesco)		sp
6	179		1	5	4	calescit	quiescit.	liquescit (liquesco)	ok
6	180	.	.	.		perscidit (perscindo)	perscindit	perscidit	≈
6	183	.		0	7	adficit (afficio)	adlicit (=allicit,0)		NB
6	185	.		46	21	alte (adv)	alti. (alo,pt)		ok
6	188	.	.	.		sint	sit		≈
6	188	.		0	7	extructa (=exstruo)	extricta (extrico,0)		NB
6	191	.	.	.		cumulata	culata		sp
6	192	.		2	20	superne (adv)	superna.		ok
6	193	.		1	4	statione	satione (satio)		ok
6	199	.	.	.		fremitus	fremitu		≈
6	205	.		36	65	color	calor		ok
6	207	.	.	.		ignis	Ignes		≈
6	208	.	.	.		flammeus est plerumque	Flammeusq. pleurumque		≈//sp
6	208	.	.	.		ollis (ille=olle=ole)	olis.		=?
6	209		138	181	27	[quin] etiam	[Quippe] enim	etenim NB	ok
6	210	.		0	3	rubeant	iubeant (iubeo,0)		NB
6	213		9 .		11	fulgore (n)	fulgere(v)	fulgere	ok
6	216	.	.	.		ingratis	ingratius		~?
6	218	.		5	41	sonitu (sonitus)	sonis (sonus)		ok
6	220		65	1238	314	ictu loca inusta	ictu et inusta	eius	=/ok/=
6	221	.	.	.		auras	auris.		≈
6	223	.		316	122	saepe	se	sae<pe>	ok
6	226	.		49	13	mobilibusque (adj)	montibusque (n)		ok
6	228		41 .		6	per saxa [per aera] (n)	per septa [domorum.] (scaepio,par	saepta	ok
6	229	.	.	.		≠	Clamor … aera.		=?
6	231		7 .		66	utei (=ut as adv)	item (adv)	item	ok
6	234	.		508	1238	et	ut		ok
6	237	.	.	.		pollens [fervore] (polleo,4)	tellens [fervere]		sp
6	241	.		160	4	tigna	igna		ok

6	242	1 .		3	lamenta	monimenta (=monumentum)	monimrenta	ok
6	246 .		4	10	[gignier e] crassis	[gigni e] classis		sp/ok
6	250	104 .		8	tum	tunc	tunc	ok
6	257 .		10	9	demissum [flumen]	dimissum [fulmen]		ok
6	257 .		42	30	[demissum] flumen	[dimissum] fulmen		ok
6	258 .		108	4	effertus (effero part.)	[et] fertus (fero part)	eCfertus (=eff-)	ok
6	269 .		4	16	plena (plenus,16)	plana, (planus,planum,4)		ok
6	272 .	.	.		habere	haecdere		sp
6	272 .	.	.		necessest	necesset.		≈
6	277 .		12	3	arto (abl. artum,3)	alto. (altum)		ok
6	281	5 .		30	gravida [aut vis ignis] (adj)	gravis [venti vis igni.]	gravis	ok
6	285 .	.	.		repente	repenati		sp
6	286	17 .		8	exprimere	Opprimere (opprimo)	opprimere	ok
6	288 .	.	.		percurrunt	pecurrunt (≠pecco)		sp
6	290 .	.	.		concussu	concussus		≈
6	291 .	.	.		uti	ut		=
6	292 .	.	.		revocari	revocare		≈
6	295 .	.	.		est	Es		≈
6	296 .		45	5	gravidam	valida		ok
6	296 .		0	42	fulmine (fulmen)	culmine (culmen,0)		NB
6	298 .		53	14	patrio	spatio		ok
6	302	5 .		35	cum(prep,conj)	Dum (adv,conj)	dum	ok
6	308 .	.	.		concepit (concepio)	concipit (12)		≈
6	309 .	.	.		ipsius	ipsis		≈
6	315 .	.	.		illi	ille.		≈
6	318 .	.	.		idonea (2)	indoneat		sp?
6	320 .		349	314	ea [quae tanto]	ex [quae tantau]		ok
6	321 .	.	.		cursu si non	cursus non		r
6	322 .		65	36	calore	colore.		ok
6	323 .		2	16	Mobilitas	Nobilitas		ok
6	324 .	.	.		percurrunt (8)	percunt		sp
6	324 .		114	1227	et	At		ok
6	335 .		37	9	deorsum (deorsum,adv,9)	deorum.		ok
6	335	25 .		18	adde	Deinde	deinde	ok
6	336 .		215	1330	plagast (+est)	plaga si		ok
6	344 .	.	.		regione	regionem		≈
6	346 .		0	108	aere	here (adv,0)	aere	NB
6	349	2 .		1	transvolat	transviat	transviat	ok
6	356 .	.	.		vincla (vinculum,8)	vincia		sp
6	357 .		46	19	apta	alta.		ok
6	359 .	.	.		veris	ris		sp
6	360 .	.	.		calore	calores		≈
6	362 .	.	.		interutraque	Inter utrasque		r
6	365 .		161	65	nubi	nobis.		ok
6	368 .	.	.		rigoris (5)	ligoris.		sp
6	369 .	.	.		ut/est?	id		=?
6	372 .		0	25	volvitur	Vovitur (voveo, 0)		NB
6	375 .	.	.		eo si tempore	eos tempore		≈//=
6	376 .	.	.		cietur	cie		≈
6	377 .	.	.		bello	bellio (≠Bellius)		sp
6	382 .	.	.		mentis	menti.		≈
6	383 .	.	.		utram se	utrame.		r
6	384 .		382	46	hinc	hic		ok
6	385 .	.	.		extulerit	eatulerit		sp
6	386 .	.	.		fulminis	fulmines		≈
6	389	25 .		10	voluptas	voluntas.	voluntas	ok
6	389 .		18	1233	quo [cuiquest]	pro [inquest]		ok
6	393 .	.	.		conscius	concius		sp
6	401 .	.	.		Iuppiter	Iuppiterras		sp
6	402 .		25	314	in eas	in aes		ok
6	406 .		25	41	vult	vivit	volt	ok
6	417 .	.	.		delubra	dilubra		sp
6	421 .		1	65	loca	ioca		ok
6	421 .		284	314	eius (pn)	plus. (adj.)		ok
6	424 .	.	.		Graiei	grali		sp
6	426 .	.	.		tam quam	tam cum	tamquam NB	r≠
6	428 .	.	.		incita	lacita (≠word)		sp
6	430 .	.	.		veniant	veniunt		≈
6	436 .	.	.		venti	vent.		sp
6	440 .	.	.		detrusit (detrudo)	detruit		sp
6	444 .		46	108	aere (n)	acri (adj)		ok
6	447 .		0	6	procellae	procellat. (procello,0)		NB
6	449 .	.	.		officere	Officeret		≈
6	452 .	.	.		coiere (≈coeo)	coire (coeo)		=
6	452	16 .		24	supero	super	super	ok
6	453	13 .		50	moris	modis	modis	ok
6	454 .		4	3	comprensa (compre[he]do),part.)	compressa (comprimo part.)		ok
6	456 .	.	.		??haec??	ea		=?
6	461	0 .		2	furvae [nubis] (furvus,adj)	fulvae [nubes] (fulvus,a)	fulvae	ok
6	465 .	.	.		maior	mmor		sp
6	466 .		2	10	[condensa queant] apparere	[condensatque arte] parere (pareo,		ok
6	467 .	.	.		videantur	videatur		≈
6	468 .	.	.		loca	lo (LO)		sp
6	469 .	.	.		sensus	sensum		≈
6	473 .		1326	1233	quo	Quod		ok
6	474 .	.	.		momine	mhomine		sp
6	475	173 .		495	ollis (ille)	omnis.	omnis	ok
6	475 .		316	1330	consanguineast (est)	consanguinea se		ok
6	476 .	.	.		fluviis	fluvis		sp
6	481 .	.	.		urget	Urgit		≈
6	481 .	.	.		super	supe		sp
6	483 .	.	.		illa	illi.		≈
6	483 .		382	19	huc	hunc		ok
6	490 .		49	16	nimbis [tempestas]	montis [tempesta]		ok/≈
6	491 .	.	.		coperiunt	Coperiant		≈
6	492 .	.	.		caulas (8)	cavias (=cauias		sp
6	496 .		10	9	demissus	dimissus		ok
6	498 .	.	.		vincam (vinco,29)	vineam		ok
6	500 .	.	.		et nubis	Et nubibus		≈
6	502 .		104	64	item	idem (104)		ok
6	503 .	.	.		consipiunt	Concidiunt		sp
6	509	0	25	72	umentia (Lachmannism?)	viventi (vivo)	umorem (humor in E) NB	ok
6	511 .	.	.		maiore	mmore		sp
6	512 .	.	.		premit	primit		sp
6	512	150 .		349	de	e	e	ok
6	514 .	.	.		calore	calorem.		≈
6	515 .	.	.		stillantque	stillante		≈
6	516 .		18	2	cera	Tela		ok
6	518	100 .		198	ubi	vi	vi	ok
6	519	370 .		114	atque tenere	At retineret	at	ok
6	520 .		228	27	cientur	fientur (fio,228)		ok
6	521 .		4	8	rigantes (rigo,8)	stillante. (stillo)		ok
6	522 .		60	381	atque (381)	aque (=aquae,60)		sp
6	523 .	.	.		umorem	humorem		=
6	523 .	.	.		redhalat	redralat.		sp
6	524 .	.	.		tempestatem inter	tempestate inte		sp/sp
6	526 .	.	.		existit	existi		≈
6	527	21 .		17	cursu	sursum	sursum	ok
6	527	21 .		17	cursuque	sursumque	sursumque	ok
6	531 .	.	.		euntis	avintis		sp
6	532 .		215	1330	perfacilest (-est)	Perfacile si		ok
6	533 .		33	228	fiant	fluant		ok
6	536 .	.	.		terram	terras		≈
6	537 .		92	3	[supera] ventosis	[super] ventis		ok
6	537 .		24	12	supera (12) [ventosis]	super (24) [ventis]		ok
6	539 .	.	.		rupes deruptaque	rupes derupes		≈/≈
6	541 .	.	.		summersaque (summerso)	summersosca		sp
6	542 .		198	312	similem esse sui (sui1)	simile esse vi		≈/=/ok
6	548 .	.	.		plaustri	plaustris		≈
6	550 .	.	.		et [ubi…via] (1238)	es [depuis…vim] (1330)	[different]	≠
6	550 .		198	52	[et ubi lapi' cumque] viai	[es depuis cumque] vim.		ok
6	554 .	.	.		vacillans	vacillas.		≈
6	558 .	.	.		urget	urges.		≈
6	563 .	.	.		????	.a.a.q.q.		????
6	563	15 .		1	meant [in eandem]	minent [in eadem]	minent	ok
6	568 .		92	198	vis [nulla refrenet] (refreno)	ventis [nulla refremet.]		ok/sp
6	582 .	.	.		versabundaque	Versabunda		=
6	584 .		5	1	diffindens	Diffidens		ok
6	586 .		1216	1159	quas	qua		ok
6	588 .	.	.		ciciderunt	cecideret		≈
6	589 .		517	2	pessum (adv)	possum.		ok

6	594 .	.	.		uti	ut [in]		=
6	600 .	.	.		complere	comple		sp
6	600 .		314	162	adque (=atque,162)	Idque (id/is,314)	atque	ok
6	604 .	.	.		subdit athuc	Subditaet hunc		r
6	604 .	.	.		parte	parti		≈
6	609 .	.	.		naturam	Natura		≈
6	616 .		198	208	sol partem	sol magnam		ok
6	621 .		162	177	ab	ad		ok
6	624 .	.	.		ventis	venti.		≈
6	626 .	.	.		crustas	crustras.		sp
6	629 .	.	.		orbi	orbis.		≈
6	632 .		152	85	maris (mare,n,85)	magis (magus,adj,152)		ok
6	638 .		108	8	detulit (defero)	tulit (fero)		ok
6	639 .	.	.		fauces	faucas		sp
6	641 .	.	.		mediocri [clade]	media [grecia de]		r
6	642 .		50	4	flammea (adj,4)	Flammae (flamma,n,50)		ok
6	648 .		3	4	dispiciendum (dispicio,v,)	despiciendum, (despicio,v)		ok
6	653 .	.	.		propositum	propositus		≈
6	653 .		9	5	plane (adv,5)	plani (planus,a;planum,n)		ok
6	658 .	.	.		obturgesict (obturgesco,)	Obturcesit		sp
6	662 .		1306	115	quia (adv,115)	qui		ok
6	663	22 .		40	orbi (22)	morbi	morbi	ok
6	667 .	.	.		concussa	concusso		≈
6	674	36 .		1306	quivis	qui [visus]	qui	ok
6	676 .	.	.		omni	omnis.		≈
6	683 .	.	.		silicum	sillicum		sp
6	687 .	.	.		contingit	contigit		≈
6	690 .		41	10	itaque	itque (eo+-que)		ok
6	695 .		10	2	resorbet	resolvet.		ok
6	698 .	.	.		[maris penitus] percocta	[mare penitus] res …		≠
6	698 .	.	.		in apertam	… cogit aperto.		≠/≈
6	701 .	.	.		vertice (vertex,9)	verteceni		sp
6	705 .	.	.		iacere	iaceret.		≈
6	708 .	.	.		nam neque	Namque		=?
6	710 .	.	.		contigit ei	contioitel.		=?
6	710 .		9	34	verum [aliquid genere]	Utrum [genere aliquid]		ok
6	716 .	.	.		etesiae	etesire		sp
6	719 .	.	.		flabra	flabro		≈
6	723 .	.	.		exoriens	Exorins		sp
6	726 .	.	.		ruit	rui		≈
6	727 .	.	.		amni	amnis.		≈
6	730 .	.	.		quod	quo		ok
6	736 .		4	4	[albas] descendere	[albas] decedere ()		ok
6	740 .		1326	1233	quo	quod		≈
6	743 .	.	.		remigi	Remigio		≈
6	746 .	.	.		substratus (4)	subiratus		sp
6	747 .	.	.		acri sulpure	ecri suiper		sp/sp
6	749 .	.	.		est/ut ?	et		?=?
6	749 .		0	233	ipso (233)	piso. (=pinso,v,0)		NB
6	754 .	.	.		Graium	glaium		sp
6	755 .		17	10	[loci vi ibus] officit	[loci opus] efficit	efficit	ok
6	755	314 .		38	[loci vi] ibus [officit]	[loci] opus [efficit]	opus	ok
6	759 .	.	.		si fit ?	sint		=?
6	759	1 .		4	mactatu (1)	mactata (4)		ok
6	761 .		0	10	apparet (10)	appare (0)		ok
6	761	0 .		228	effiant	[e] fiant	fiant	ok
6	762 .	.	.		Puteis (2)	poteis		sp
6	764 .		3	3	inferne (adv)	inferna		ok
6	767 .		6	34	vera	vere (adv)		ok
6	770 .		0	161	cuiusque	culusque (culus,0)		NB
6	771 .		64	1191	quae	eque (med. aeque,64)		ok
6	777 .		64	96	auris (32+64)	auras (64)		ok
6	778	1 .		23	adactu	tactu.	tactu	ok
6	780 .		0	15	tristia	tristitia (0)		NB
6	787 .	.	.		necare	netare.		sp
6	788 .		5	265	[ideo] terris	[indeo] tris		[sp]/ok
6	791 .	.	.		acri	acris.		≈
6	793 .	.	.		spumam ut qui ??	pumos qui		sp
6	798 .		4	3	labefactant	labefaciant		ok
6	799 .	.	.		cunctare	cunctere		sp
6	800	1	3	1330	[et] laveris	efflueris	fueris NB	ok
6	801 .		150	900	in	de		ok
6	803 .	.	.		cerebrum	celebrum		sp
6	803 .	.	.		aquam	aqua		≈
6	804 .	.	.		fervidior	fervidaser		sp
6	804	24	3	4	domus	domnus (=dominus)	domans (domo,v) NB	ok
6	805	5 .		6	viri	vini	vini	ok
6	806 .	.	.		ipsa	ipso.		≈
6	808 .	.	.		argenti	argento		≈
6	811 .	.	.		metalla	metulla		sp
6	813 .	.	.		audisve (-ve)	audire		≈
6	815 .	.	.		necessis	necessest. (necesse+est)		≈
6	818 .	.	.		loca alitibus	locam alitbus		≈/sp
6	818	314 .		1238	ea	et	et	ok
6	820 .	.	.		quadam	quodam		≈
6	820 .		0	53	spatium	patium (pator,-oris,0)		NB
6	825 .	.	.		vitae	vita		≈
6	831 .	.	.		terramque	teramque		sp
6	832 .		46	411	hic [linquatur]	hinc [linquitur]		ok/sp
6	840 .	.	.		frigidior	Que frigidior		≠
6	841 .	.	.		semina si quae	semi siqua.		≠/≠
6	841 .		0	6	rarescit	Arescit (aresco,0)		NB
6	842	1	2	14	propere (adv)	proprie (adv)	proprii (n)	ok
6	846 .		900	508	ut	in		ok
6	849 .	.	.		frigidus	Frigidas		≈
6	851 .		27	6	raptim	partim.		ok
6	857 .		24	19	supter (=subter)	super		ok
6	858 .	.	.		perquoquere	Perquocere		sp
6	858 .	.	.		satiare	soclare		sp
6	860 .		1330	127	suum	sum		ok
6	862 .	.	.		rara [tepet]	Para [tenet]		sp
6	862	0 .		60	[rara] tepet	[Para] tenet	tenet	ok
6	864	16 .		35	umbris	undis.	undis	ok
6	865 .		41	28	penitus	sonitus		ok
6	868 .	.	.		=?	laticis		=?
6	868	18 .		44	saporem	vaporem.	vaporem	ok
6	870	4 .		36	gliscente (glisco)	miscente (misceo)	miscente	ok
6	874 .	.	.		radiis	radis		sp
6	877 .		9	10	dimittat	demittat		ok
6	878 .	.	.		nodosque	nobosque		sp
6	879 .	.	.		frigidus	Fridigus		sp
6	880 .		0	23	iacit	lacit 0		ok
6	887 .	.	.		multa	muita		sp
6	888	47 .		95	Propterea (47)	Preterea (=praeterea,95)		ok
6	889 .	.	.		conciliari	conciliare		≈
6	890 .	.	.		marist [?Aradi]	marisparat		sp
6	892 .		12	15	praebet	praeter		ok
6	894 .	.	.		dulcis	dulcit		sp
6	896	1233 .		1191	quo	que (=quae)		ok
6	897 .		3	8	taedai corpore	deda corpora (dedo,didi,imp)		ok
6	898 .		1306	113	quia	qui		ok
6	899	0 .		60	tepentis	tenentes.	tenentes	ok
6	900 .	.	.		lumina	lumines (lumen)		sp
6	907 .		1	27	lapis	lapsi		ok
6	908 .		1361	970	quem	Quam		ok
6	909 .	.	.		ortu	ortus.		≈
6	912 .	.	.		quinque	Qui neque		r
6	913 .	.	.		demissos	demisso		≈
6	913 .	.	.		iacterier [auris]	iacterer [auras.]		≈
6	913 .		64	96	[iacterier] auris (96)	[iacterer] auras. (64)		ok
6	915 .	.	.		vinclaque	vinciaque		sp
6	916 .	.	.		permananter	permanater		sp
6	916	2 .		1	pervolat	pervalet	pervalet	ok
6	919 .		0	2	ambagibus (2)	ambaginibus (0)		ok
6	922 .		0	63	mitti [spargique]	miti [sparcique] 0)		NB
6	923 .		60	1191	quae	aque (=aquae)		sp
6	925 .	.	.		fluviis	fluvis		sp
6	925 .	.	.		ab sole	a sole		=
6	928 .	.	.		venit	vent		=/=
6	931 .	.	.		requies	requis		≈
6	937 .		1	1	claret	clare. (adv)		ok

6	938	.		.		.		ad res	ardes.		r
6	940	.		.		.		firmare	firmara		sp
6	942		20	.			1	superne (adv)	superna. (adj)	superna	ok
6	954		3	.			150	Galli	caeli	caeli	ok
6	956	.		.		.		tempestate	tempestatem		≈
6	957	.			0		4	iura (4)	iurae (Jura,0)	iure	ok
6	958	.		.		.		corpori' nexu	corpore nexum.		≈/≈
6	962	.		.		.		=?	quo		=?
6	964	.		.		.		extructasque	Extructas		=
6	969	.		.		.		calore	calori.		≈
6	971	.		.		.		ambrosiae	ambrosias		≈
6	971		0	.			7	[vere et nectari'] linctus	[vero et nectare] tinctus. (tingo)	tinctus	ok
6	972	.			0		2	amariu' [frondeat esca] (amaror)	marius [frondeac exscet] (0)		NB
6	973	.		.		.		amaracinum	maraeinum		sp
6	977	.		.		.		iucunda	civunda		sp
6	986	.			19		217	alioque	aloque		sp
6	991	.		.		.		praeterea [manare]	Praetere [manere]		sp
6	991	.			41		15	[praeterea] manare	[Praetere] manere		ok
6	992	.			160		16	lignis	ignis		ok
6	1000	.		.		.		omnis	omnes.		≈
6	1001	.		.		.		pelliciat vim	peliceatum		sp
6	1006	.		.		.		ferri	ferre.		≈
6	1007	.			4		508	utque (=ut)	utqui.		ok
6	1011	.		.		.		natura	naturae		≈
6	1012		25	.			76	[quo] ducitur	[quod] dicitur	dicitur	ok
6	1012	.			1326		1233	quo [ducitur	quod [dicitur		ok
6	1014	.		.		.		anulus	anullus		sp
6	1018	.			1238		349	e	et		ok
6	1020	.		.		.		plagis	plagit		≈
6	1021	.		.		.		sponte	Sporte		sp
6	1022		508	.			64	utei (=ut)	item	item	ok
6	1023	.		.		.		iuvetur	iuvatur.		≈
6	1025	.			198		152	magis	magnis		ok
6	1026	.			0		108	aer	Er (0)		NB
6	1032	.		.		.		ventus	ventis.		≈
6	1033	.		.		.		locatus	locacatis.		sp
6	1040	.		.		.		ille	illo		≈
6	1046	.		.		.		scaphiis	scapiis		sp
6	1047	.		.		.		ab	a		=
6	1059		1238	.			114	et	At	at	ok
6	1062	.		.		.		interutrasque	Inter utrasque		r
6	1062	.		.		.		locata	loata		sp
6	1064	.		.		.		eam	eum		≈
6	1064	.		.		.		flumine	flumina		≈
6	1067	.		.		.		singillariter apta	se singulariter aptem		/≈/≈
6	1068	.		.		.		vides	vide		≈
6	1068	.		.		.		colescere	coolescere (=colescere)		=
6	1069	.		.		.		uno	una.		≈
6	1071	.		.		.		vincla	vincia.		sp
6	1076	.		.		.		renovare	nenovara		sp
6	1077	.		.		.		eiuere	eiuere (≠ejulare)		sp
6	1078	.		.		.		res aurum	res auro		=/≈
6	1079		108		0		25	aerique aes plumbo	Aeraque plumbo (aera,0)	aes	NB//=
6	1089	.			108		228	fieri	ferri		ok
6	1091	.		.		.		cladem	cradem		sp
6	1100	.		.		.		coortae	coorta.		≈
6	1109	.			36		65	colore	calore.		ok
6	1115	.		.		.		Aegypto	aegypta		sp
6	1119	.		.		.		alienum	aelenum.		sp
6	1121	.		.		.		ut	Ve (-ve)		≠
6	1122	.		.		.		graditur conturbat	graditus conturbas		≈/≈
6	1124	.		.		.		reddatque	reddetque		≈
6	1125	.		.		.		pestilitaque	estilitasque.		sp
6	1132	.			0		2	balantibus (2)	calantibus (0)		sp
6	1135	.		.		.		coortum	corumptum. (≠conr-,corrumpo,		sp
6	1135	.			2		2	ultro	uitro (=vitro)		ok
6	1138	.		.		.		morti' ferai	mortiferae.		r
6	1139	.		.		.		Cecropis	cecropit (≠v)		sp
6	1141	.		.		.		veniens	venies		≈
6	1141		40	.			4	morbus	ortus.	ortus	ok
6	1143	.		.		.		omnei	omnem.		≈
6	1148	.		.		.		ulceribus	viceribus		sp
6	1148	.		.		.		coibat	coibet.		≈
6	1150	.			0		23	tactu (n)	tacta. (adj,0)		NB
6	1165	.			107		517	potuis	totius (totus)		ok
6	1166	.		.		.		ulceribus	viceribus		sp
6	1167	.			198		508	ut est	vi est		ok/=
6	1171	.		.		.		vertere	Verteret		≈
6	1171	.		.		.		frigora	figore		≈
6	1177	.			85		26	mali	mari		ok
6	1178	.			1		2	lymphis	nymphis		ok
6	1180		0	.			5	[ac] nuntia [morbis] (nuntia)	ardentia [morbis,] (ardeo,p)	ardentia	ok
6	1186	.		.		.		spiritus	spiritum.		≈
6	1187	.		.		.		umor (≠humor)	umum. (≠humus)		sp
6	1189	.		.		.		raucas	rauca		≈
6	1189	.		.		.		tussi	tusse.		sp
6	1192	.		.		.		supremum	suremum		sp
6	1192	.		.		.		tempus	temps.		sp
6	1195	.			17		1	inhorrescens rictum	inhoretiacet rectum		sp/ok
6	1195	.			0		2	tumebat	mebat. (0)		NB
6	1196	.		.		.		rigidi	rigida		≈
6	1198	.			7		153	aut	Avi		ok
6	1199	.		.		.		=?	ut est		≠
6	1200	.		.		.		ulceribus (ulcus)	Viceribus		sp
6	1205	.		.		.		qui taetri	cul taetris		sp/≈
6	1212	.			5		3	his incesserat (incedo)	iis incusserat (incutio)		≈/NB
6	1213	.		.		.		etiam	eiam		sp
6	1217	.			4		25	exeiret (≠exiret)	exciret (excido)	exiret	ok
6	1220	.		.		.		? tristia ?	tia		=?
6	1221	.		.		.		exeibant	Exicbant		sp
6	1225		102	.			93	cernebant (cerno)	certabant (certo)	certabant	ok
6	1228	.			1		24	tueri (24)	turi. (1)		sp
6	1235	.		.		.		apisci	apiscit.		≈
6	1238	.		.		.		visere	utsere		sp
6	1240	.		.		.		poenibat	Poenibus at		r
6	1241	.		.		.		incuria	incura		sp
6	1249	.		.		.		lectum	iectum		sp
6	1250	.		.		.		morbus	morbo.		≈
6	1251	.		.		.		talei (poetic?)	tale.		≈?
6	1260		2	.			13	labes [quem]	languens (langueo)	languens	ok
6	1261	.			0		6	morbida (adj)	morbide (adv, 0)		NB
6	1262	.		.		.		conplebant	condiebant		sp
6	1262		0	.			6	astu	aestus.	aestu	ok
6	1264		6	.			6	protracta (protraho)	prostrata (prostreno)	prostrata	ok
6	1265	.			1		30	iacebant	tacebant.		ok
6	1270	.		.		.		pelli	pellis		≈
6	1271	.		.		.		ulceribus	Viceribus		sp
6	1271	.		.		.		sordique	sordeque		≈
6	1272	.		.		.		replerat	rellerat.		sp
6	1274		60	.			41	tenebat	manebat.	manebant	ok
6	1282	.		.		.		subitae	subitaf		sp
6	1285	.			14		124	faces (facio)	fauces		ok
